# Activation of the Internal Surfaces of Polymer Tubes with Argon Plasma Through Hollow Cathode Effect

**DOI:** 10.3390/polym18192416

**Published:** 2026-10-03

**Authors:** Jang-Hsing Hsieh, Jyh Wei Lee, Chuan Li, Stephen Hsieh, Shin Yo Shen, Nima Bolouki

**Affiliations:** 1 Department of Biomedical Engineering, National Yang Ming Chiao Tung University, Taipei 300093, Taiwan; 2 Center for Plasma and Thin Film Technologies, Ming Chi University of Technology, New Taipei City 243303, Taiwan; jefflee@mail.mcut.edu.tw (J.W.L.); no1dogtwo@gmail.com (S.Y.S.); 3 Department of Materials Science and Engineering, University of California, Riverside, CA 92521, USA; hsiehguy@gmail.com; 4 Plasma Technologies, Central European Institute of Technology—CEITEC, Brno University of Technology, Purkyňova 123, 61200 Brno, Czech Republic; nbolouki@gmail.com

**Keywords:** plasma surface modification, inner tube surface, hollow cathode effect, hydrophilicity

## Abstract

The focus of this study is on generating hollow cathode discharge (HCD) plasma inside three types of polymer tubes, resulting in the activation of the tubes’ inner surfaces. To achieve this goal, both theoretical calculations and experiments were carried out to prove that the adopted methodology was feasible. By applying optical emission spectroscopy, with line ratio and modified Boltzmann’s plot methods, the plasma sheath could be estimated and applied to the prediction of HCD generation. To verify whether HCD prevails in the tube, the various power inputs (70~260 W), tube diameters (3~7 mm), and working pressure (0.1 and 0.4 torr) were selected. Accordingly, it was confirmed that the tube diameter must be greater than twice the plasma sheath in order to form HCD, which would then activate the surface. Ar gas was used with a capacitive-coupled RF plasma system. PET, PU, and PTFE tubes were used in this study. The tubes’ inner surfaces were characterized with XPS and water-contact-angle (WCA) analysis. The results also show that once HCD is generated inside the tubes, these tubes’ inner surfaces could become hydrophilic, and the values of WCA might decrease by 40°, 45°, and 70° respectively for PET, PU, and PTFE, due to the combined effects of the enhanced ion bombardment and radical oxidation. XPS studies show that, for PET and PU, C-O and, particularly, C=O bonds increase significantly. For PTFE, the C-F bonds decrease, while carbon–oxygen bonds increase. Overall, the surface activation is thought to be enhanced by ion bombardment attributable eventually to the hollow cathode effect (HCE).

## 1. Introduction

Surface modification with low-temperature plasma is one of the most popular research topics. So far, many plasma-based surface-related processes have been developed and applied in industries. On the other hand, polymers are known for their flexibility, low density, low cost, and ease of manufacturing. However, their surface properties often cannot satisfy the demands on low gas transmission, high biocompatibility, high wettability, etc. [1]. Many studies have reportedly sought to improve the surface chemistry or morphology of polymers. Unfortunately, the use of chemical treatments may cause changes in polymer structure [2] that affect their overall characteristics. Also, these methods are normally not environmentally friendly. Hence, suitable surface modification by cold plasma is required to achieve the desired surface properties [3,4,5,6].

Tubes made of polymers are widely applied in human daily life and in critical engineering fields (e.g., fluid transport in biomedical fields). In biomedical industries, surface activation of tubes is often required. To succeed in this attempt, the most critical challenge is to generate plasma inside the tube and subsequently modify the tube’s inner wall. However, so far, only minor efforts have focused on surface modification within the tube, including surface activation or functionalization. Only a few papers have reported the application of cold plasma for the surface activation [7] or functional coating on tubes’ inner surface [8,9]. Li et al. [9] applied argon/oxygen plasma by discharge and reported that the plasma generated inside the tube could change the state of the tube’s inner surface. Among all the approaches adopted for the modification of polymer surfaces, cold plasma is a well-known process to achieve the needed surface properties without the use of extra physical and chemical reactants. This is highly desirable for the modification of polymers being applied for biomedical applications, or many other uses [10]. Another significant benefit of plasma treatment is that it is considered a green process, relying on types of discharge, treatment time, and characteristics of the applied gases [7,8,9,11].

The effects of low-pressure plasma processes on water contact angle (WCA) or surface energy of various substrates have been studied for a long time. The characteristics of plasma depend on the gas input to the plasma discharge. For example, O_2_, H_2_, N_2_, and CF_4_ produce O, H, N, and F radicals, respectively [12]. Sharm et al. [13] reported that WCA was observed to change from 80° to 40° after plasma activation for 600 s in Ar plasma for polyimide (PI). In this study, surface activation is most likely induced by the bombardment of Ar ions. Getty and Zhao [14] reported that if the PI surface is treated with remote Ar, N_2_, and O_2_ plasma, the substrate would not be exposed to ion bombardment. The results show that the exposure of the substrate to Ar and N_2_ plasma only slightly reduced the WCA, while oxygen plasma caused a large decrease due to the presence of O radicals. In the latter case, the WCA of PI is reduced from 60° to 5°. Egitto and colleagues [15] found that the WCA of PI was reduced from 70° to 20° after direct exposure of the substrate to He/O_2_ plasma for only 50 s. In their conclusion, the surface energy of the polymer substrate can be significantly increased by the treatment with plasma due to the generation of O radicals, which should be beneficial to improve the wettability of hydrocarbon-based polymers [16]. Furthermore, many efforts have been made to functionalize polymer surfaces by plasma processing. The functionalized polymer surfaces by plasma may contain -COOH, -OH, -NH_2_, -COOR, -COR, -CFx, -Br, -SH, and thiophene-based units [12]. Great interest in these functionalized surfaces comes from their fundamental applications in modern biomedical applications, including interlayers to promote metal modification adhesion [17], biocompatibility [18], antimicrobial modification [19], or drug release [20].

In this study, low-pressure cold plasma was applied to activate the inner surfaces of PET (polyethylene terephthalate), PU (Polyurethane), and PTFE (Polytetrafluoroethylene) tubes with Ar plasma, as plasma can be generated through hollow cathode discharge (HCD). A similar approach developed in this study can be applied to plasma functionalization of polymer tubes.

PET is a kind of thermoplastic polyester. It is one of the most widely used polymers currently in various industries. In life science, the surface states of PET could be tailored according to blood–polymer or cell–polymer interaction [21]. Plasma processing could be applied directly to control these interactions. Additionally, plasma can be applied to increase or decrease reactive sites, aiming at immobilizing or loading bioactive components, which may include antithrombotic and anticoagulant agents [22].

PU is a polymer known for its well-accepted mechanical properties, wear resistance, flexibility, durability, and biocompatibility, along with light weight and smooth finishing [23]. Although PU has all these advantages, for certain applications where low WCA and excellent adhesion are needed, the application window of PUs is quite narrow. Specifically, its high WCA would usually induce poor adhesion [24,25]. The adhesion problem can be traced back to the fact that it has a shortage of usable polar functional groups. Therefore, further surface activation is required for quite a large number of applications [26,27]. Plasma processing was proved to be able to incorporate various polar functional groups on PU’s surface. Improved hydrophilic behaviors could hence be achieved. [28].

PTFE is a kind of thermoplastic polymer, mainly with C-F bonds. It has a melting point close to 600 K, before which the mechanical properties can be maintained. Accordingly, it has been very popular in many industries. Its chemical inertness, attributed to C-F bonds, however, will cause very poor adhesion of any additional substance or thin film coatings. To solve this problem, many research topics have focused on the improvement of wettability. It has been a major technological and scientific task for the past decades. However, it is one of the most difficult polymers to activate [29]. So far, there are many studies on the activation of PTFE by plasma. The predominant mechanism leading to a hydrophilic surface is still not well understood. In some studies, it is proposed that bond scission during treatment is required. This might be caused by ultraviolet radiation and ion bombardment rather than direct functionalization with reactive oxygen species [30].

**Figure 1:**
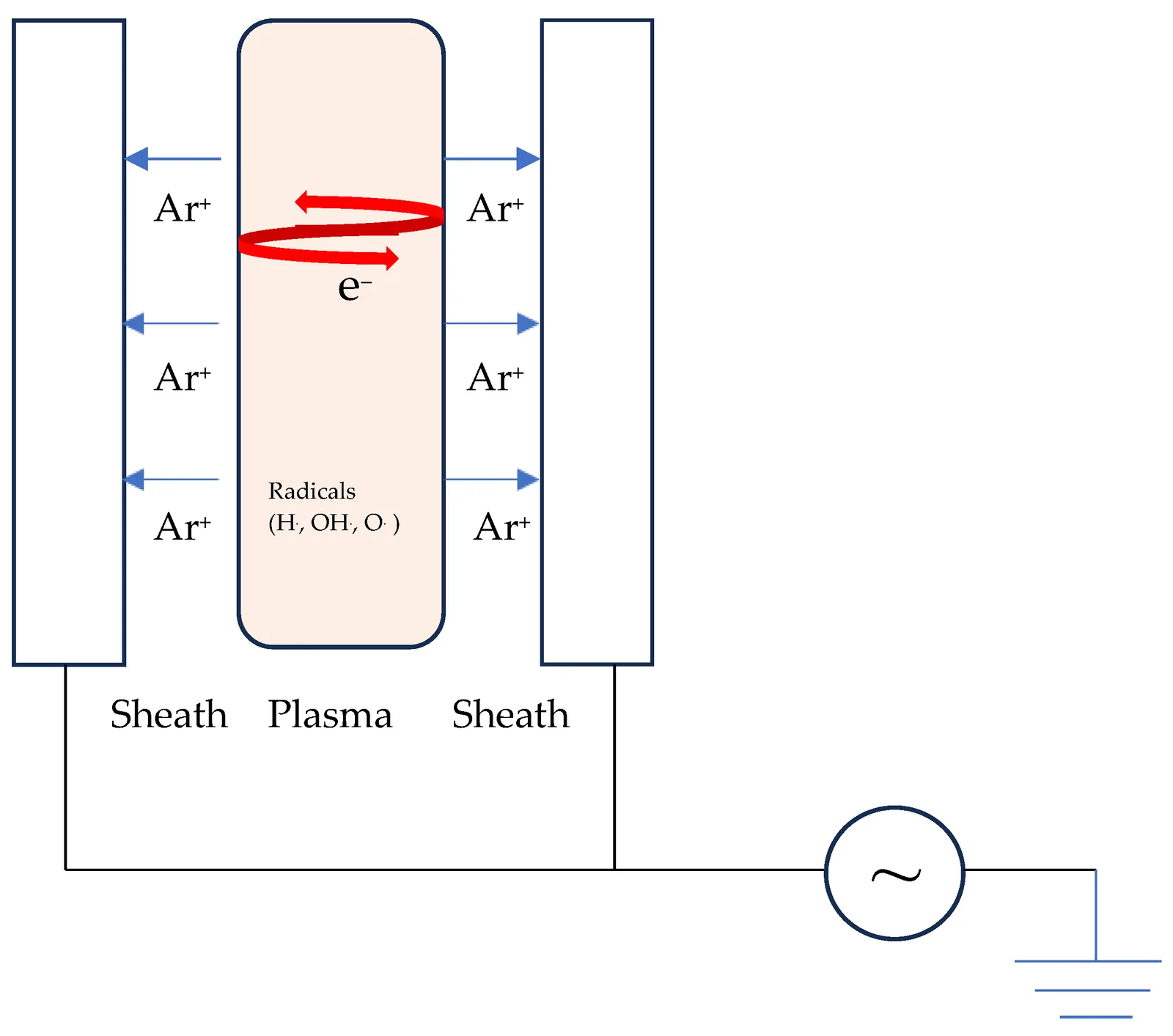
Schematic drawing of an RF hollow cathode and possible discharge. The pendulum movement of oscillating electrons is responsible for the occurrence of HCD.

To date, many plasma systems, including atmospheric-pressure and low-pressure plasmas (APP and LPP), are used to treat various polymers. However, there are only a few studies on the plasma modification of tubes’ inner surfaces, and none of these reports applied the theory of HCD and understood how it can be generated to proceed with the process. An HCD device normally consists of a cathode with a hollow structure (ex: aperture, hole, tube) [31], as illustrated in Figure 1. Compared to conventional CCP discharge, hollow cathode discharge is outstanding due to higher concentrations of electrons and ions, as well as radicals (i.e., high plasma density). These properties will contribute to the phenomenon known as the hollow cathode effect (HCE) [31,32].

As shown in Figure 1, HC and oscillation movement of electrons between repelling electric fields originating from the two plasma sheaths are illustrated. In general, electrons emitted from the cathode surface accelerate toward the opposite side due to the electric field through the sheath and are bounced back. As a result, these electrons oscillate across the narrow band between the two opposite inner walls of the electrode due to the repelling fields of the opposite plasma sheaths, and it can cause more frequent collisions with the gas in the glow. Ions may accelerate toward the cathode walls and hence cause severe ion bombardment that would directly result in bond breakage. Furthermore, photoemission of electrons is also possible due to UV light irradiation. This may further increase the ionization rate and therefore plasma density [33]. Inside the tubes, ions may work to break up some of the bonds on the polymer’s surface. These fractured bond sites are then chemically available to form polar bonds with radicals, thereby creating an activated polymer surface.

In HCD, the thickness of the plasma sheath normally decreases with the increase in power or gas pressure, which means the electric field in the plasma sheath may be increased. Thus, the electrons generated on one cathode surface would accelerate towards the negative glow and are retarded only by the opposite cathode sheath. This would allow electrons to perform several ionizing collisions. In addition, the ion bombardment of positively charged ions on the cathode surface would cause secondary electron emission, which in turn causes more ionization. Without question, metastable atoms are also generated in the processes [34,35,36]. In sum, these HCE mechanisms depend significantly on the operating parameters, the characteristics and pressure of gas used in the hollow region, the cathode material, electrical circuit, and HC geometry. [37]. Regarding the effect of HC width/diameter on plasma density (electron density), it has been reported that the plasma density increases with the decrease in width/diameter until it reaches the threshold value.

In the following, Table 1 summarizes some representative examples of surface activation on various polymers with conventional capacitively coupled plasma (CCP) and unconventional high-density plasma technologies. Overall, most of the literature proposed that surface activation is related to the increase of oxygen concentration and the formation of C-O or, more effectively, C=O bonds. In the case of PTFE, the removal of fluorine atoms, followed by oxygen bonding, was normally critical [38,39]. Furthermore, according to this table, ion and radical density and the related ion bombardment may play critical roles in activating polymer surfaces.

The purpose of this study is to study and apply low-pressure plasmas for the activation of tubes’ inner surfaces. When tubes are attached to the cathode (power electrode), they can be considered as hollow cathodes (HC). For HCD to be generated inside a tube, it is thought that the tube diameter should be greater than twice the plasma sheath. Otherwise, plasma will not be initiated inside the tube. Subsequently, the tube’s inner surface will not be activated [45]. The previous study [45] studied the effects of MMA monomers on plasma characteristics. Hence, the generation of HCD could be affected, and the accumulation of PMMA polymerized coatings might or might not be induced. In the present experiment, PET, PU, and PTFE tubes with various diameters were used for the HCD activation study. The HCE mechanisms are similar between these two. However, the applications are quite different. Furthermore, transferred plasma jets will be applied for comparison reasons in future studies. One of the major works in this study is to verify whether the hollow cathode effect (HCE) prevails in the modification process, with the variation in tube diameter and power input. To carry out the experiment, the plasma sheath was calculated according to the results of optical emission spectroscopy (OES). From the OES results, the temperature and density of electrons in the plasma could be obtained with line ratio and modified Boltzmann’s plot methods [46,47]. The plasma sheath was then estimated and finally compared with the experimental results to confirm the formation of HCD.

## 2. Experimental Procedures

### 2.1. Plasma Activation Source and Process

**Figure 2:**
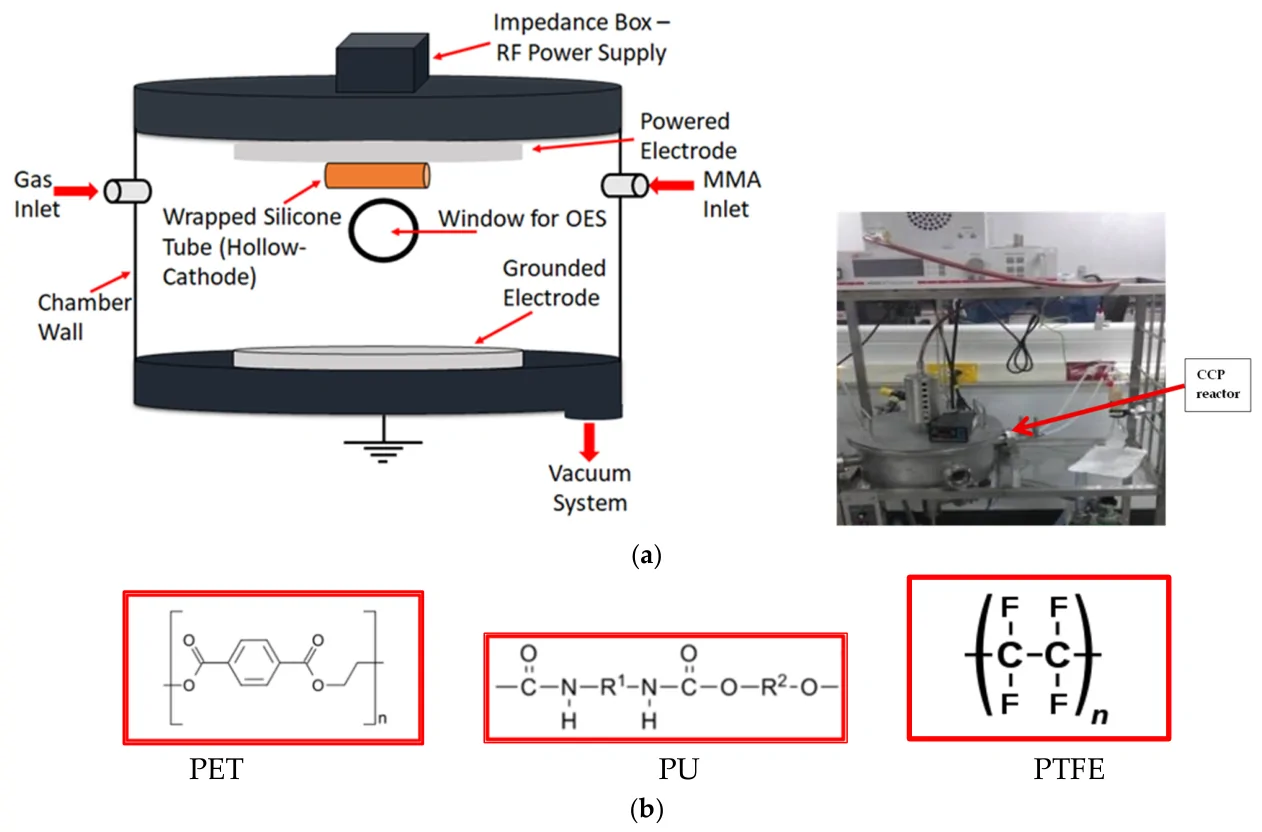
(**a**) Schematic drawing and real image of the capacitively coupled RF plasma (CCP) setup for HCD plasma activation, (**b**) chemical formulas for three tube materials.

**Figure 3:**
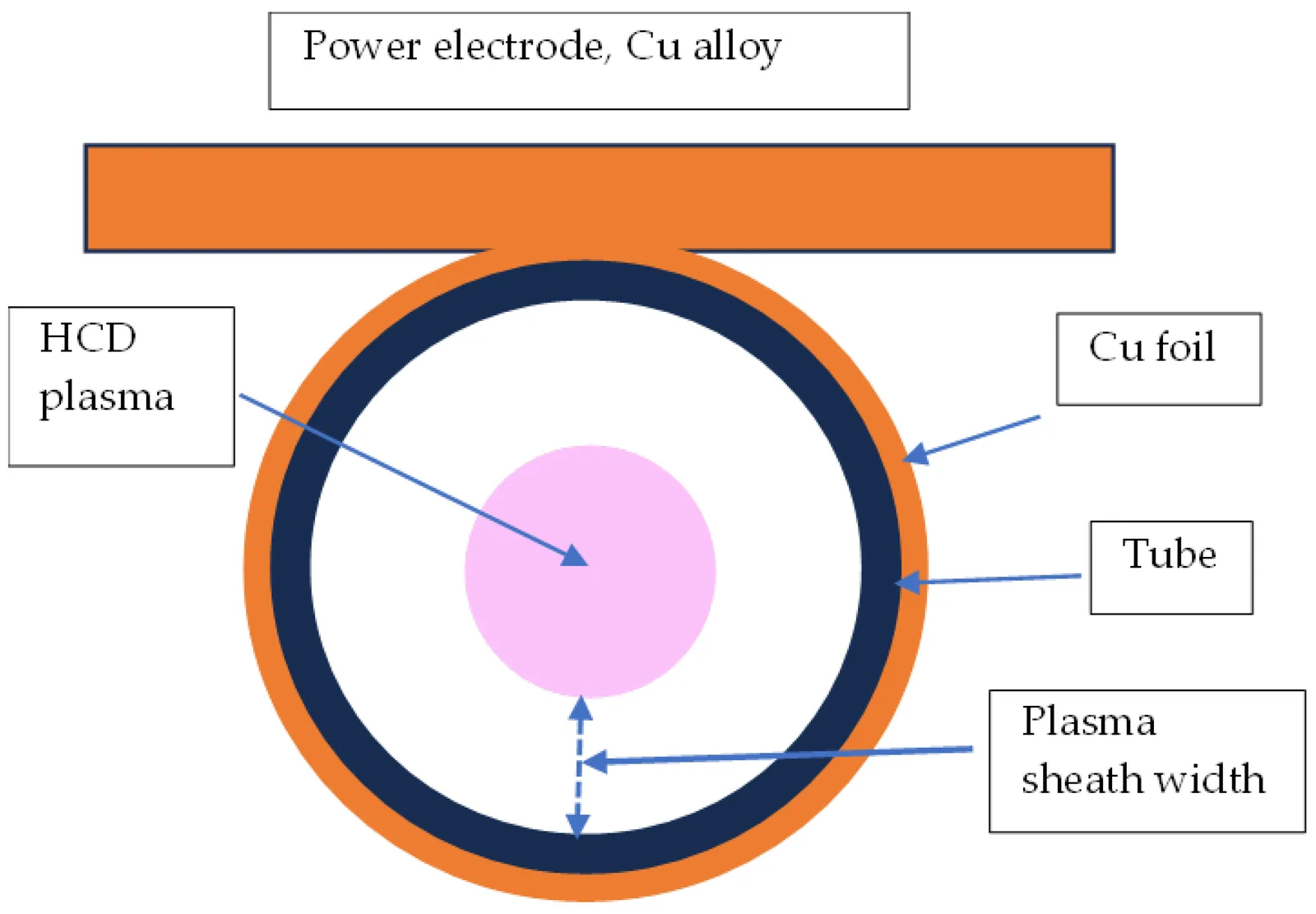
The cross-sectional drawing of a tube attached to the power electrode (made with Cu alloy).

The schematic of the home-made RF reactor is displayed in Figure 2. The RF discharge was generated in a grounded stainless-steel vacuum chamber having an internal volume of 7.5 L, with dimensions of 35 cm (dia.) × 15 cm (height). Two parallel circular plate electrodes with a diameter of 24.5 cm were placed 60 mm apart. The powered electrode was capacitively coupled to an RF generator (13.56 MHz). The bottom electrode was grounded. The negative bias was formed naturally on the power electrode. The chamber was pumped down using a mechanical pump to an ultimate pressure of 1 × 10^−2^ Torr and then filled with Ar gas (99.999%, Cingfong Gas Industrial Co., Taoyuan, Taiwan). A 7 cm window enabled the observation of the discharge. The tubes (lengths of 10 and 15 cm and inner diameters of 3, 4, 5, 6, and 7 mm) were attached to a powered electrode after the tubes were wrapped with copper foil to induce HCE. The cross-sectional drawing of a tube attached to the power electrode is illustrated in Figure 3. To make sure the tube was in contact with the power electrode, copper tape was used to press down the tube onto the electrode. The tubes were cleaned with DI water in an ultrasound cleaner (LEO Ultrasonic Co., LTD., New Taipei City, Taiwan) for 20 min, then blown dry with nitrogen. Experiments were carried out at various power inputs and tube diameters to understand how they affect plasma characteristics. For the detection of excited species in plasma, an OES was employed. The optical collecting lens was attached to the chamber quartz window during the deposition process and focused on the inside region of the tubes. The spectrometer captures the plasma spectra inside the tubes and chamber. More detailed information can be found in the literature [45]. In this study, PET (VPET Plastic Industrial Co., LTD., Taichung, Taiwan), PU (Taiwan PU Corp., Kaohsiung, Taiwan), and PTFE (Fluo-Tech Industrial Co., LTD., Taoyuan, Taiwan) tubes with various diameters were used for the HCD activation study. Table 2 shows the material characteristics of the selected tubes [48,49,50].

The polymers containing aromatic rings (e.g., PET and PU) exhibited a high level of oxygen uptake. This can be driven by the reactions of ring-opening attributed to ion bombardment. Afterward, hydroxyl groups may be formed on the polymer surface and enhance hydrophilicity.

### 2.2. Verification of Plasma Generation Through HCE

Table 3 shows the process parameters selected for the verification of plasma generation inside the tube, while Table 4 shows the process parameters for the modification of the tubes’ internal surfaces, followed by the characterization by WCA and XPS.

#### Estimation of Plasma Sheath

As mentioned in the previous sections, to modify the inner surface of a polymeric tube, a plasma needs to be generated inside the tube. It is therefore required that the tube diameter (Td) should be greater than twice plasma sheath (D_s_), i.e., Td > 2D_s_. The plasma sheath can be estimated as in the following [51].

D_s_~η^2/3^ × λ_D_ (1)

Here,

D_s_ = thickness of plasma sheath

λ_D_ = Debye length

η = e × (V_P_ − V_B_)/(k × T_e_)

Sheath potential (plasma potential) is given by

V_s_ = (k × T_e_/2e) × ln (m_e_/(2.3 × m_i_)) (2)

Here,

V_s_ = sheath Potential

m_e_ =mass of electron in kg

m_i_ = mass of ions in kg

k = Boltzmann’s Constant in JK^−1^

T_e_ = Electron Temperature in eV

n_e_ = Electron density in cm^−3^

The Debye length can be estimated by the following equation:

λ_D_ = (ε_0_k_B_T_e_/e^2^n_e_)^1/2^ = 743 × (Te/n_e_)^1/2^ (cm) (3)

where *T_e_* is the electron temperature in eV and *n_e_* is the electron density in cm^−3^ [52]. For the plasmas generated in the present study, a common value of λ_D_ is within 2 × 10^−2^ to 4 × 10^−4^ cm for *n_e_* = 1 × 10^9^–1 × 10^13^ cm^−1^ and *T_e_* = 1–2.5 eV. For chamber pressures between ~0.13 up to ~1333 Pa (roughly 1 mTorr to ~10 Torr) cold plasma’s λ_D_ is between 0.1% and 1% of the electrons’ collisional mean free path [52].

The above-mentioned approach for the estimation of plasma sheath should be considered as the upper limit of its value since the *n_e_* inside the tube is normally greater than outside the tube in the chamber. During the measurement of OES spectra, inevitably, some part of chamber plasma characteristics may be collected, especially for small diameter tubes. However, with the decrease in diameter, the plasma density should increase until the threshold diameter is reached.

Here, the modified Boltzmann method [46] and the line-ratio method [47] were applied for *T*_e_ and *n*_e_ estimations, respectively. For the estimation of *T*_e_ using the modified Boltzmann method, the Ar emission peaks of 696.1, 714.8, 731.7, and 750.4 nm were used. For the line-ratio method, the intensities of Ar emission lines of 357.2 nm and 750.4 nm were applied.

### 2.3. Material Characterization

**Figure 4:**
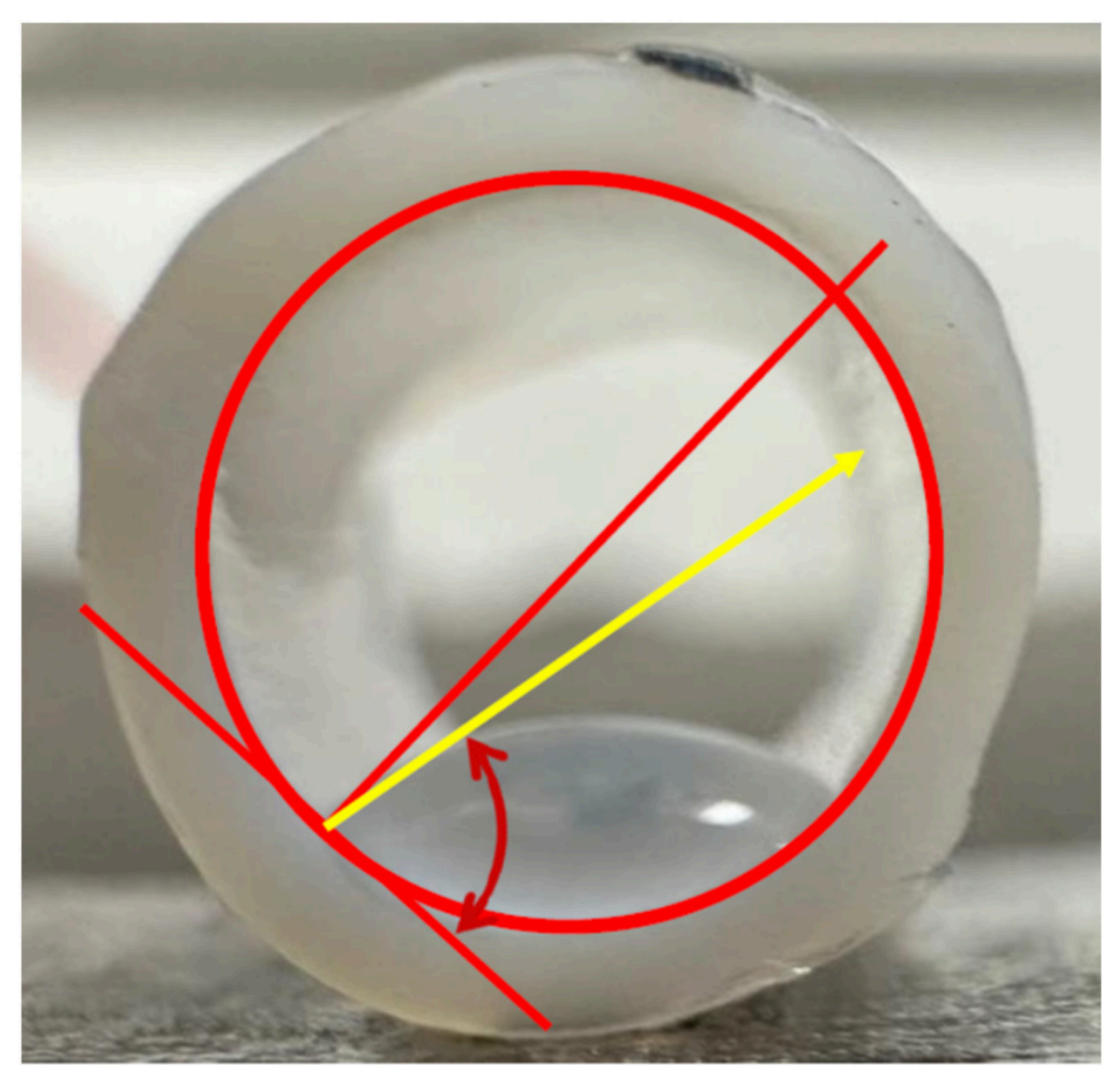
Illustration of WCA on the tube’s internal surface (The arrow stands for the measured WCA).

A contact angle measurement system (homemade) was used to check the WCA of the tubes’ internal surfaces before and after plasma processing, as functions of tube diameter and plasma power. Prior to WCA and XPS experiments, the tube was cut into equal sections of 1 cm. For WCA measurement, a one mL dispenser (syringe) was used while the water droplet volume was controlled at 0.1 μL. Each sample was measured three times. Error bars represent the difference between the smallest value and the largest value. The measurement of contact angle is illustrated in Figure 4. The measurement was carried out right after HCD treatment. The binding energies of C, O, N, and F elements on the tubes’ inner surface were examined by an X-ray photoelectron spectrometer (XPS, PHI 5000 VersaProbe III, ULVAC-PHI. Inc., Tokyo, Japan). The calibration of peak positions used the C1s peak (284.6 eV). For the calibration, the gold sample surface was measured to obtain the carbon peak position. The carbon signal on the gold surface may originate from the environment. The pass energy was 15 eV. This will give a fixed resolution of 0.9 eV. Prior to analysis, the tube samples were cut open to form a small piece with dimensions of 5 × 5 mm. The chemical binding situation of those critical elements could be determined from the spectra.

### 2.4. Diagnostics of Plasma Using OES

In the system, there is a quartz viewport that enables the passage of various lights generated inside the plasma and captured by OES. The collected light intensity may contain those generated inside and outside the tube. The collected OES spectra were later analyzed using the above-mentioned approaches. The spectrometer (AVANTES, AVASpec-2048L, Apeldoorn, The Netherlands) had a spectral range within 200–1100 nm. The spectral resolution was 1.4 nm. In every OES experiment, the collecting lens was attached to a clean quartz viewport to capture the emission spectra in the chamber, with the focus at the tubes’ internal space during the plasma surface processing. The diameter of the optical fiber is 3 mm. The distance between the tubes’ opening and the viewing port is 5 cm. For every new measurement, the sensor head was moved around until the maximum intensity was obtained. The approaches used to obtain electron density and temperature, and the subsequent plasma sheath, can be found in the previous literature [45].

## 3. Results and Discussion

### 3.1. Verification of Plasma Generation Through Hollow Cathode Effect

**Figure 5:**
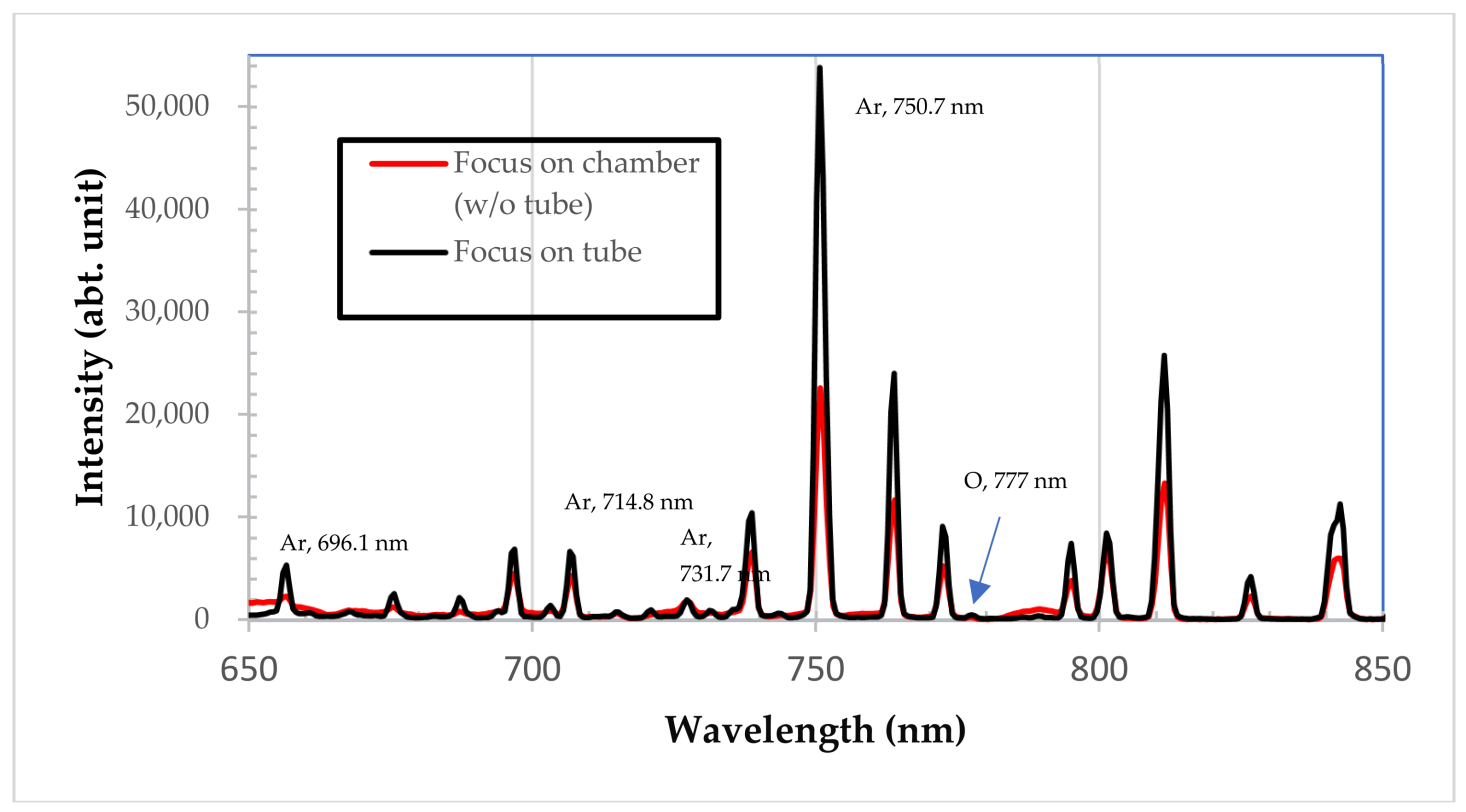
Two representative spectra covering Ar-I emission peaks and minor O peaks, one is obtained from the plasma inside a 6 mm tube, another one is obtained from the chamber plasma, generated at 260 W and 0.4 torr.

The OES spectra were used to calculate *T_e_* and *n*_e_. Figure 5 shows two representative spectra; one is obtained from the plasma inside a 6 mm tube, and another one is obtained from the chamber plasma generated at 260 W and 0.4 torr. It can be observed, according to the peak at 750 nm (Ar), that the HCD plasma has a density around three times that of chamber plasma. As shown in Table 5 and Table 6, by increasing the RF power from 70 to 260 W, the electron density (*n*_e_) increases with the decrease in electron temperature (*T*_e_), with vacuum pressures of 0.1 and 0.4 torr. λ_D_ is known to be proportional to the square root of *T*_e_ and 1/*n*_e_ [45]. Thus, λ_D_ decreases with increased RF power, as seen in both tables. Therefore, the sheath thickness would decrease with the increase in RF power, as sheath thickness is proportional to λ_D_. As described previously, *T*_e_, *n*_e,_ along with λ_D_, provide critical information for the estimation of sheath thickness. The immediate effect of increasing RF power on plasma characteristics of HCD can be described as follows: (1) The increased excitation and ionization rates of Ar atoms in plasma were the main reason for the increase in plasma density. (2) In the HCD plasma, the plasma intensity is increased due to collision impact on Ar atoms by the oscillating electron. When RF power increases, the ionization and excitation rates are enhanced, and *n*_e_ therefore is increased. (3) For similar reasons, *T*_e_ would decrease due to the increased collision frequency of electrons. As shown in Table 5 and Table 6, it can be observed that the value of *n*_e_ increases with the decrease in *T*_e_ when RF power increases. It is also important to know that the working pressure plays a critical role in terms of plasma characteristics because the mean free path (MFP) would decrease with the increase in working pressure. In this case, *n*_e_ will increase while *T*_e_ will decrease.

**Figure 6:**
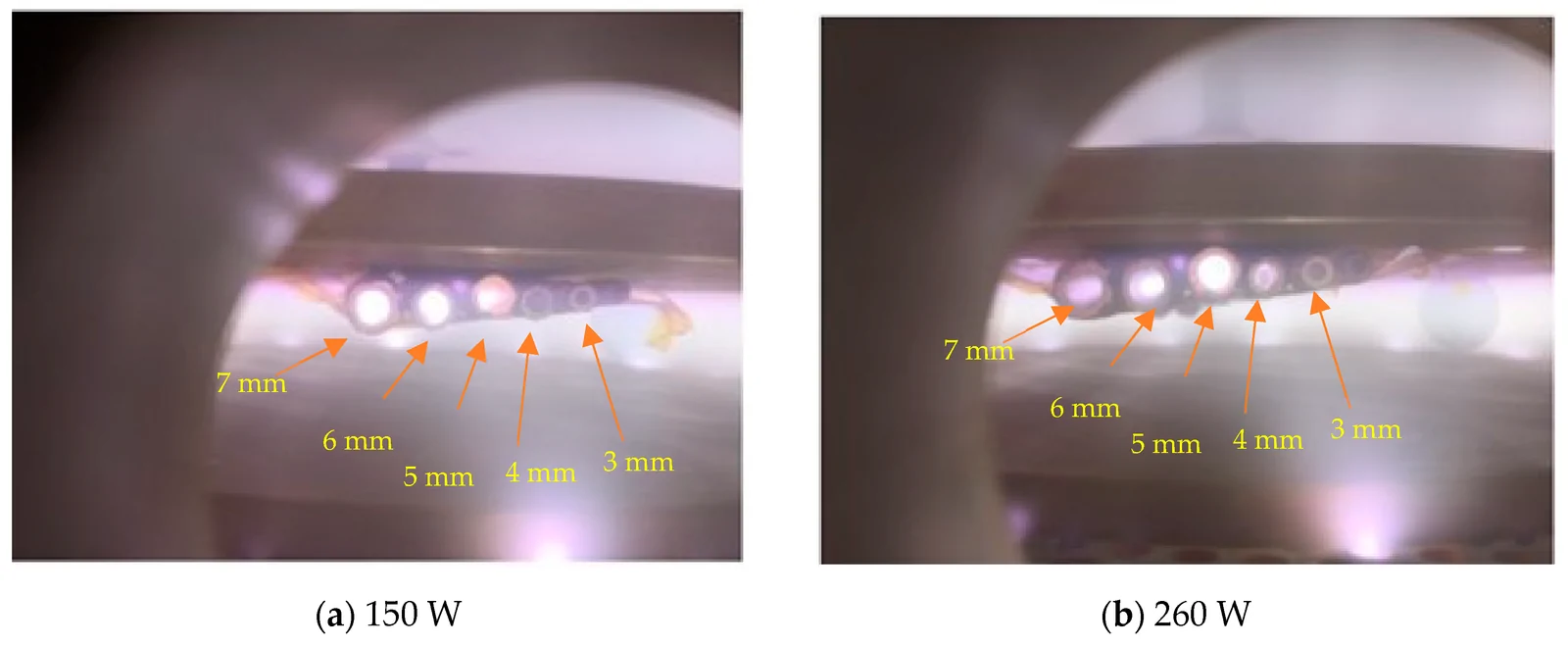
Generation of HCD plasmas after RF power was turned on, with the variation in tubes’ inner diameters and RF power at (**a**) 150 watt, and (**b**) 260 watt. (working pressure = 0.4 torr).

As mentioned before, the inner tubes’ diameters were varied with the change in process parameters. Tubes’ inner diameters between 3 and 7 mm were selected for the initiation study of plasma inside the tubes. If the value of sheath thickness is equal to or greater than the internal diameter of the tube, the HCD plasma will not be initiated inside the tube (i.e., no glow discharge). Figure 6 shows the cross-sectional images of tubes with RF power set at 150 W (Figure 6a) or 260 W (Figure 6b). According to Figure 6, the experiments with RF power set at 150 W could not generate stable plasma inside the tube when the diameter was either 3 mm or 4 mm. This is because the sheath thickness was greater than the inner radius of the tube. However, if the RF power was increased to 260 W, the tubes with an inner diameter of 4 mm or bigger could generate HCD plasma inside the tubes. The results are consistent with the results shown in Table 6. With the power set at 150 W, the sheath thickness was calculated as 2.38 mm, which implies that the tubes with diameters less than 4.76 mm would not be able to generate plasma inside the tube. On the other hand, if the power is set at 260 W, the tubes with diameters less than 3.88 mm would not generate plasma inside the tubes.

The broadening of the sheath thickness will inevitably result in the sudden drop of the electric field along the radial direction of the tube. Eventually, there would be no plasma generation inside the tube for some samples with small diameters. Without plasma generated inside the tubes, the inner surfaces of the tubes could not be modified.

Table 5 and Table 6 also show the values of *T_e_*, *n_e_*, and plasma sheath thickness under different power and chamber pressure of Ar plasma. The results show that when the power increases from 70 W to 260 W, *T_e_* decreases, and *n_e_* increases, and the sheath thickness also decreases. According to the hollow cathode theory, for a tube with a 4 mm diameter, only one condition can result in the generation of plasma inside the tube. This condition would be the one that applies 260 W under 0.4 torr in chamber pressure. In both tables, the last column shows the values of T_e_/D_s,_ which represent the electric field across the sheath. It can be observed that the electric field strength increases with the increase in power. Furthermore, the maximum ion bombardment energy is proportional to T_e_, according to Equ. 2. However, ion bombardment flux is related to plasma density.

### 3.2. Water Contact Angle Analysis

The wettability (hydrophilicity) of polymers is normally reflected by WCA qualitatively. Extensive work has been carried out on various polymers using different plasma systems. In one example, Koh et al. [53,54] used an ion-assisted process to study surface activation on various polymers in reactive environments. The results showed that the formation of polar functional groups, including C=O, (C=O)-O, C-O, etc., is critical in effective surface activation of polymers.

**Figure 7:**
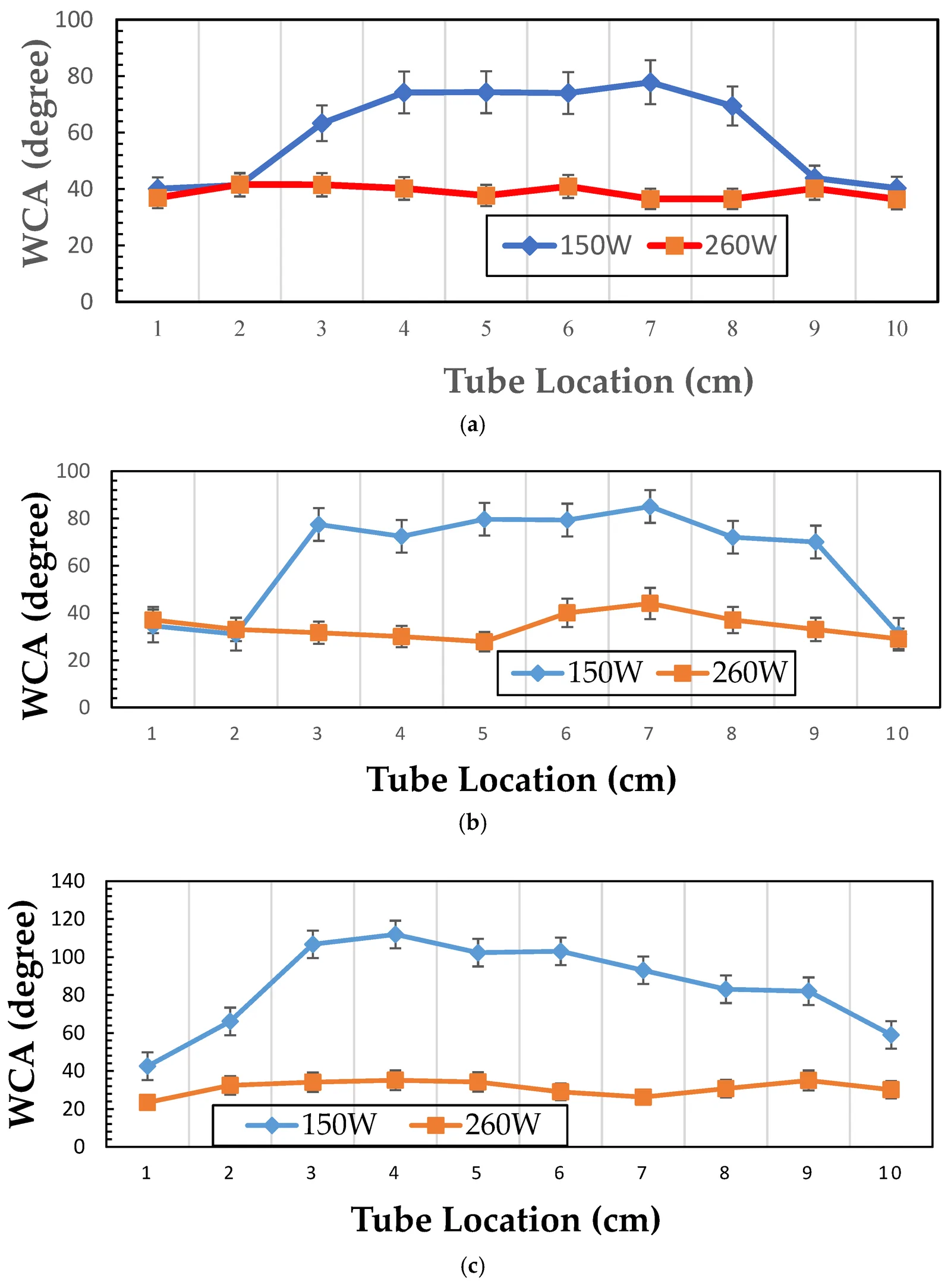
WCA of the processed tubes at different locations along the tube, with (260 W) and without (150 W) HCD plasma. (tube length = 10 cm, tube inner diameter = 4mm): (**a**) PET, (**b**) PU, (**c**) PTFE.

**Figure 8:**
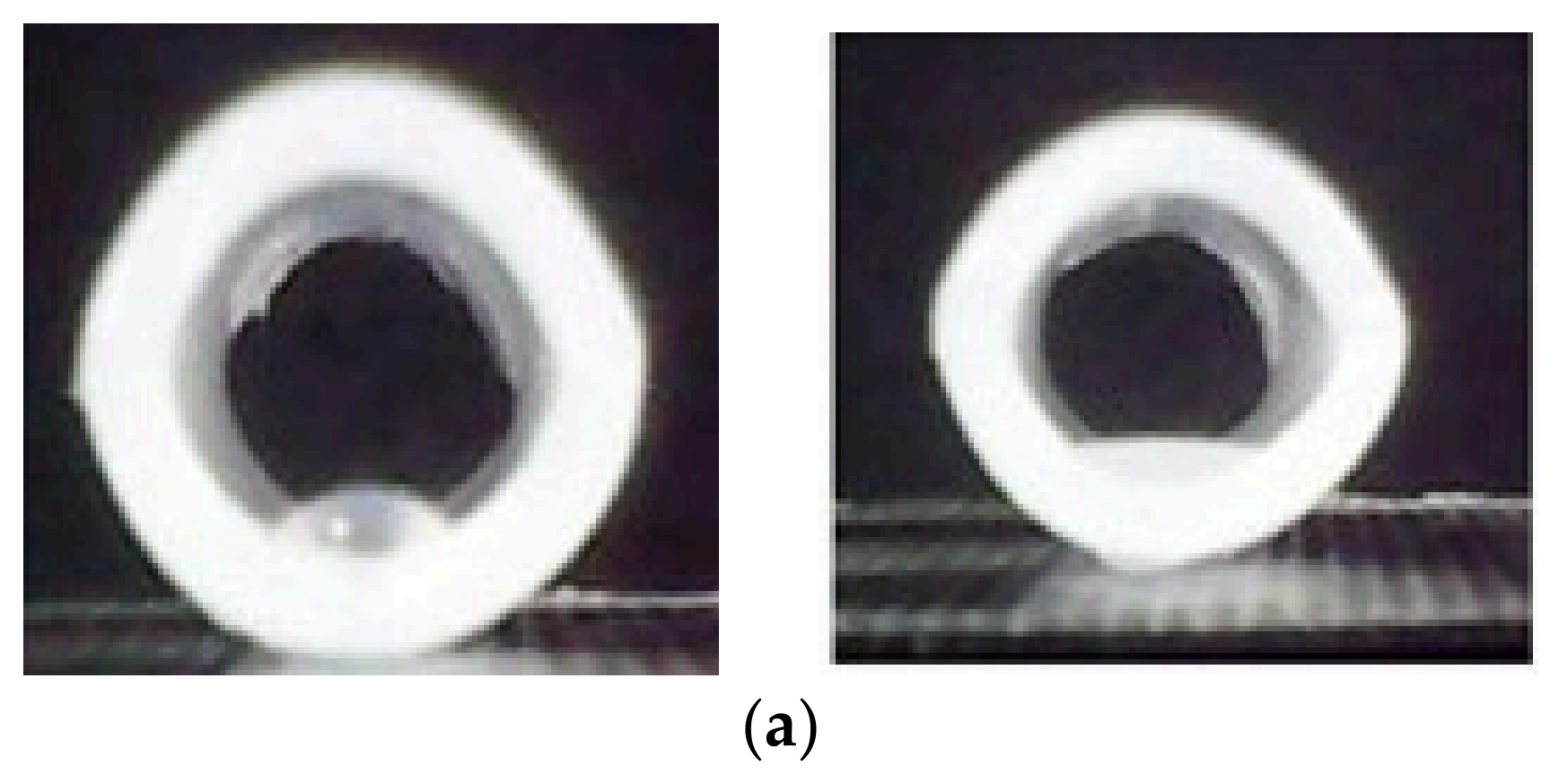

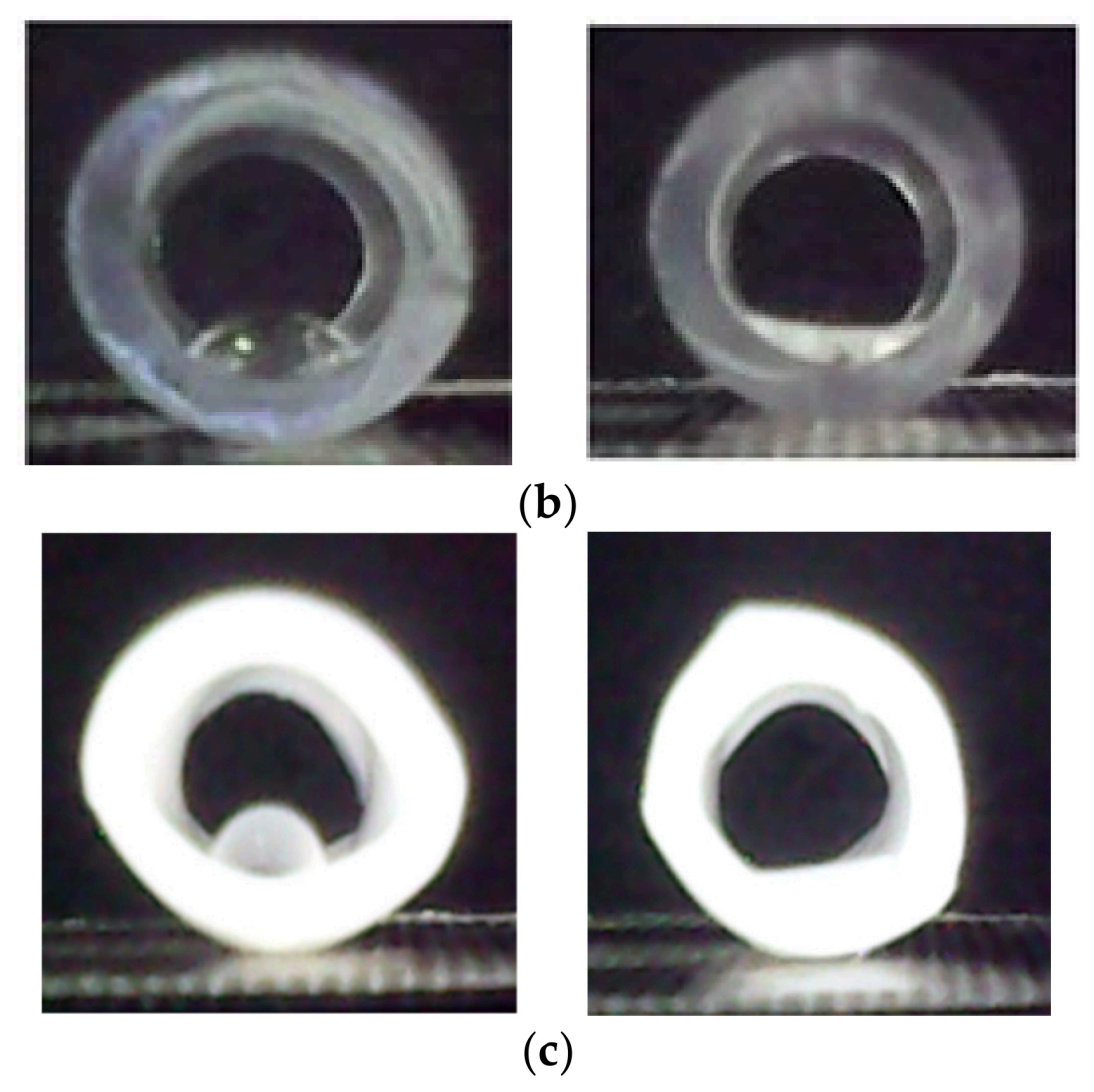
Images of water droplets on the inner surface of untreated (**left**) and HCD plasma-treated (**right**) tubes, (**a**) PET, (**b**) PU, and (**c**) PTFE. (inner diameter = 4 mm).

The WCA values for original PET, PU, and PTFE are 74, 80, and 101, respectively. Figure 7 shows the values of WCA as a function of tube location, with input powers at 150 W or 260 W for PET, PU, and PTFE tubes. Whether the tube had generated HCD plasma could be observed through the viewing port. In this part of the experiment, all the tubes had a fixed internal diameter of 4 mm, and all were treated in Ar plasma of HCD for 5 min. As seen in Figure 6, the plasma could not be generated inside the tubes with the input RF power set at 150 W. The WCA on the internal surfaces is at the highest near the center region. For example, PET had a contact angle of 73.1°, while PU had 79.6°, and PTFE had 103.3°. With RF power set at 260 W, HCD plasma can be generated inside these tubes, and hence the surface can be activated. Under these circumstances, the water contact angles were 38.7°, 33°, and 30.2° for PET, PU, and PTFE, respectively. These results can echo the previously calculated plasma sheath. The sheath thickness is 2.38 mm with input power at 150 W, and it is 1.94 mm with input power at 260 W. Figure 8 shows some images of the water droplets on the samples taken from the tubes’ center regions. According to Figure 7, although there was no plasma generated in the tubes, with input power set at 150 W, a certain degree of surface activation can be observed near both opening ends, particularly on PET samples. These results are thought to be due to the diffusion of the oxidative radicals, such as OH, H, and O, into the tubes, which may cause chemical oxidation. For PET, this phenomenon is much more obvious, since the role of ion bombardment is not as important as that for PTFE or PU. In the study, although Ar was used as the working gas, the ultimate vacuum was about 10 mtorr. Hence, a quite large number of water and air molecules were involved in plasma activity. This would cause the formation of various radicals with tremendous oxidative power.

**Figure 9:**
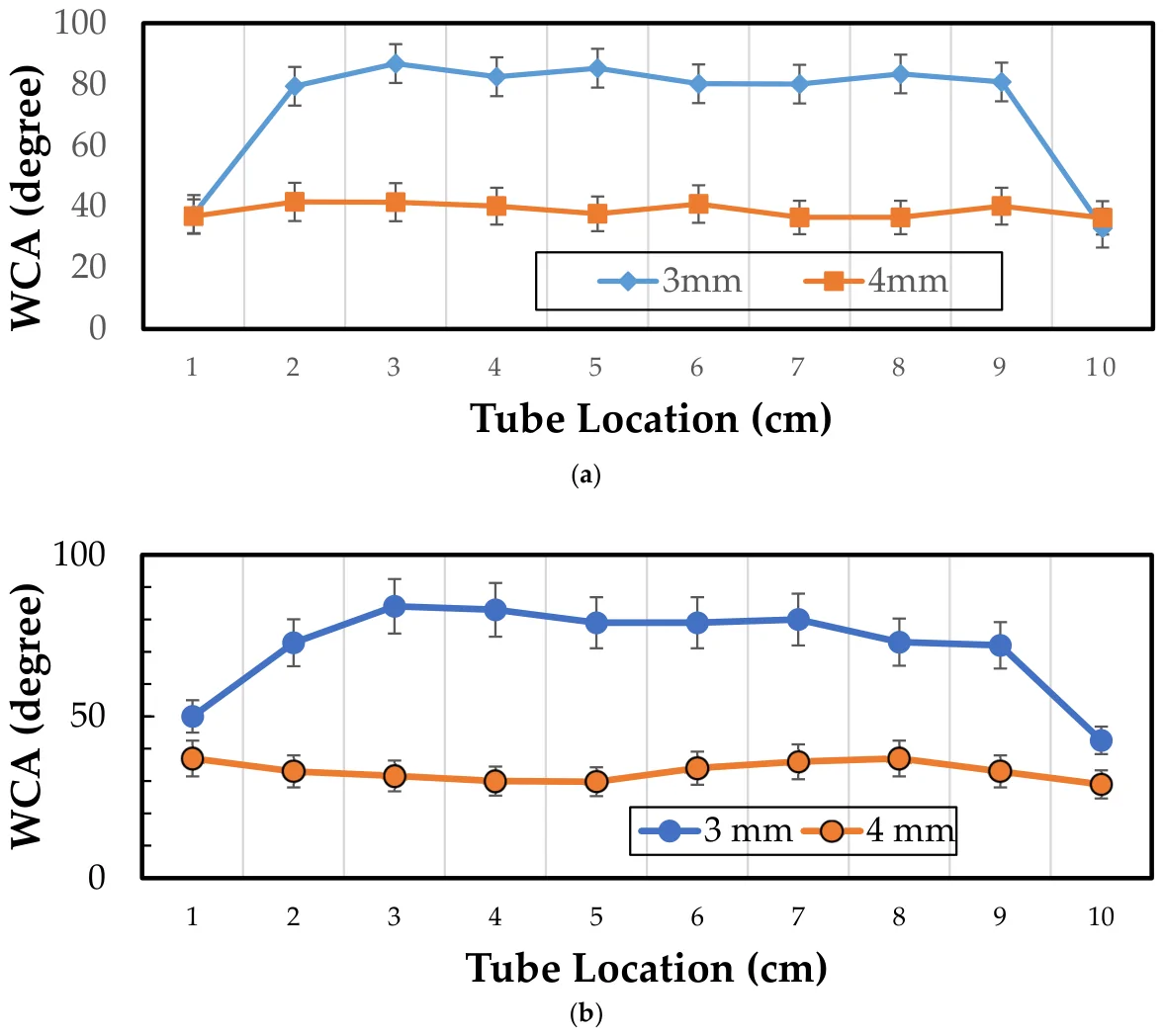

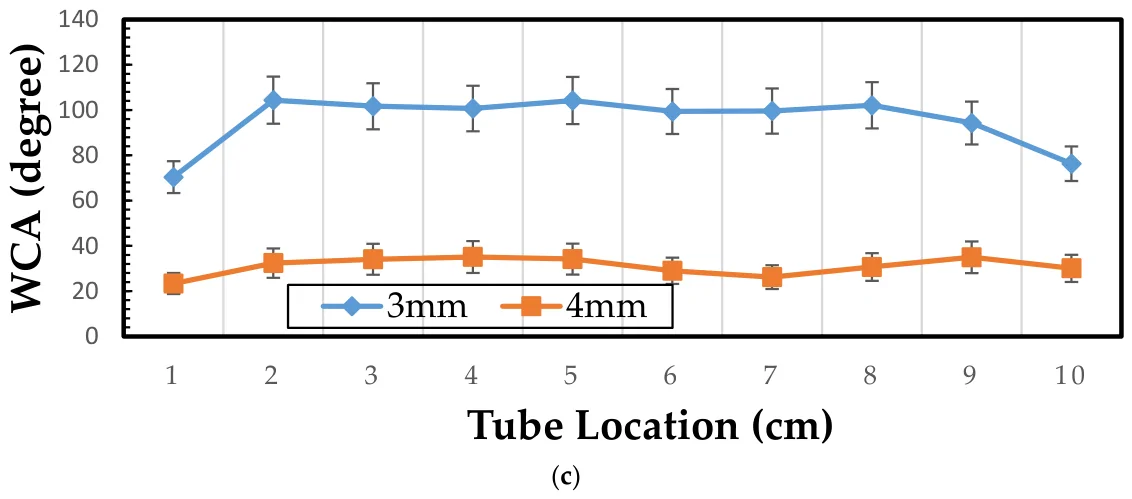
WCA of the processed tubes at different locations along the tube, with internal diameters of 3 mm (without plasma) and 4 mm (with HCD plasma). (tube length = 10 cm, RF power = 260 W): (**a**) PET, (**b**) PU, (**c**) PTFE.

Figure 9 shows the WCA values of tubes as a function of tube location, with internal diameters of 3 mm or 4 mm, under Ar plasma of HCD for PET, PU, and PTFE tubes. The experiments were carried out with a fixed power of 260 W. It was observed that the WCA of the tubes with an internal diameter of 3 mm was about 85.3°, 79.8°, and 104.2° at the center point. This is because no plasma was generated in 3 mm tubes. For 4 mm tubes, the plasma was generated inside the whole tubes. The internal surfaces were hence activated. The WCAs of the tubes were about 38.7°, 33°, and 30.2°, respectively. For 3 mm tubes, at the locations near both ends, the contact angle tends to be lower. This would be due to the penetration of plasma, and further due to the diffusion of oxidative radicals, although it is not as significant as in Figure 7. Since the diameter is smaller (i.e., 3 mm), it is expected that the diffusion distance of radicals would be shorter.

The improvement of wettability, as reported by Valerio et al. [55], can be explained as the synergistic effects of plasma radicals and UV/VUV (vacuum ultraviolet) radiation. In the present study, the radicals may come from water and air molecules remaining in the chamber, since the experiments were carried out in a vacuum chamber that was pumped out only by a mechanical rotary pump. Based on this, it is possible to tell the difference between two kinds of plasma processing that may enhance different mechanisms in surface modification: (i) plasma activation with radicals, and (ii) plasma treatment with plasma species that may cause bond-scission first and the subsequent depletion of fluorine or carbon from the surface. PET activation is a typical example of the first case. For PTFE and even PU, the mechanism that causes bond breaking of C and F atoms arises from ion bombardment, which is the typical result of HCE. After bond scission, some reactive species would readily interact chemically with the polymer surface, forming a polar surface. This result will cause the formation of unstable fragments containing carbon, fluorine, and oxygen, which may increase the surface energy.

It was reported previously that, for most F-free polymers, the hydrophilic surface can be commonly obtained by plasma sustained in pure O_2_ or a mixture of O_2_ with an inert gas. The difference between F-free polymers and PTFE is explained as follows. For PTFE, each carbon atom in PTFE is bonded to two F atoms. The C–F bond strength is known to be one of the strongest among all chemical bonds. Therefore, the bond breakage requires high-energy ion bombardment. This may occur in HCD. Additionally, F has the highest oxidation potential compared with all other elements. Therefore, the substitution of F atoms with O or OH radicals is not thermodynamically possible. Hence, the existence of O radicals from plasma does not necessarily lead to the substitution of F on the surface of PTFE. The bombardment energy and momentum carried by energetic Ar species (particularly, Ar ions) are sufficient to break C-F bonds. As described by Carbone et al. [38], the interactions simultaneously induced by reactive O species and the ion-bombarded surface could cause the formation of volatile CxFyOz molecules, which may desorb from the PTFE surface eventually. Therefore, the net reaction may be like reactive ion etching.

With the presence of water molecules, it is possible that the additional hydrogen radicals would enable the breakage of C–F bonds and the creation of dangling bonds on the surface. The best results in terms of WCA were reported recently by Nguyen et al. [56]. The WCA value changed from 118° to 4°. In this study, they applied an inductively coupled RF discharge with a power of 100 W. Several gases were used. The results showed that O_2_ and Ar plasmas did not show obvious improvement since the WCA remained at 112° and 110° with both plasmas. By using plasma generated in Ar mixed with NH_3_ or H_2_O, a significant reduction in F concentration near the surface was found. Accordingly, hydrophilic functional groups containing O and N were detected on the surface of PTFE. As a result, Ar ions may contribute to breaking bonds, such as the C-F bond, while radicals, such as O, may form C-O and C=O bonds. This further explains the importance of HCE, which may provide large amounts of energetic Ar ions and radicals.

**Figure 10:**
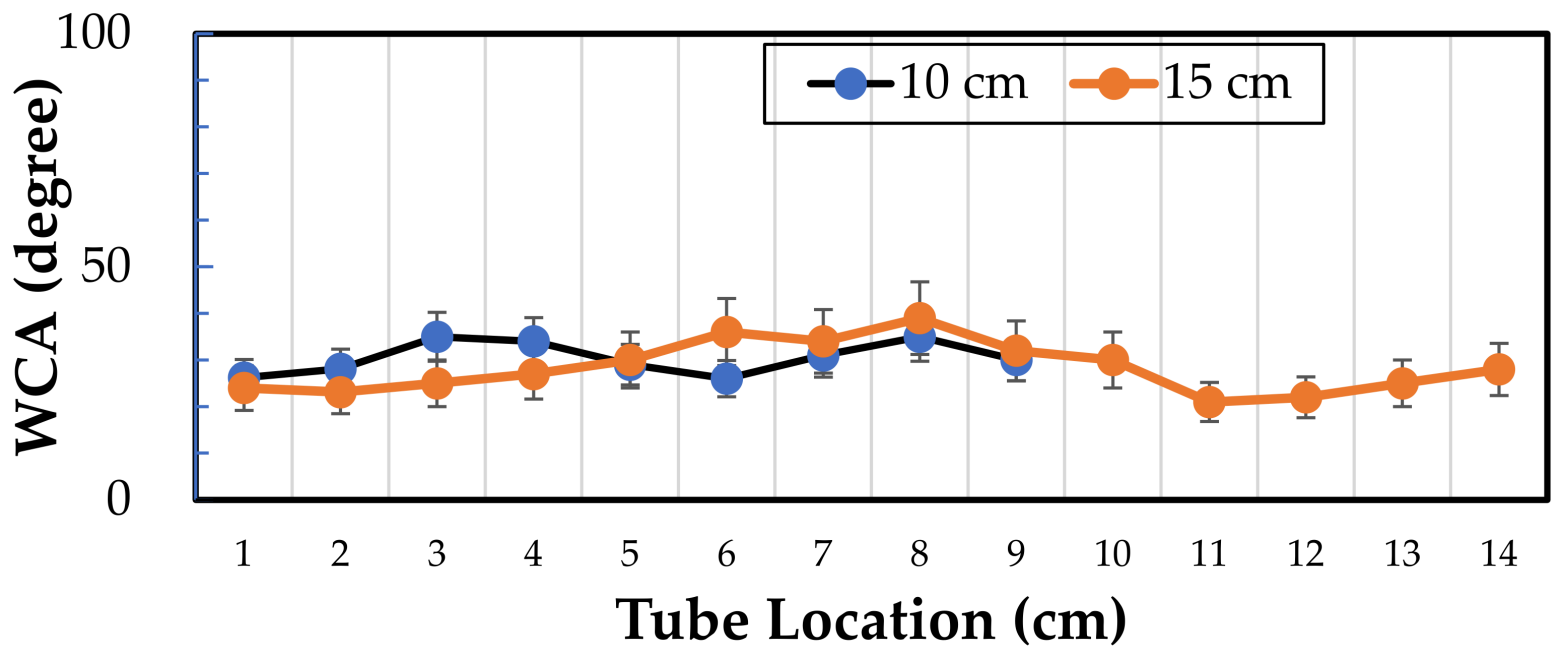
WCA of the PTFE tubes at different locations along the tube after HCD plasma treatment, with lengths of 10 and 15 cm. (RF power = 260 W, inner diameter = 4 mm).

Figure 10 shows two plasma-treated PTFE tubes with a diameter of 4 mm, but with different tube lengths. It is seen that the length would not affect the modification results, since HCD plasma can be generated inside the tube no matter how long the length is. Recently, Chen et al. [57,58] applied TAPP (transferred atmospheric pressure plasma) to treat the inner surface of a PTFE tube and found that a uniform and effective surface activation with any length can be easily obtained by moving the tube along its axis. In the present study, the uniform treatment of tubes can also be realized if HCD plasma can be generated inside the tube.

### 3.3. Chemical Bonding Analysis of the Tubes’ Internal Surfaces

**Figure 11:**
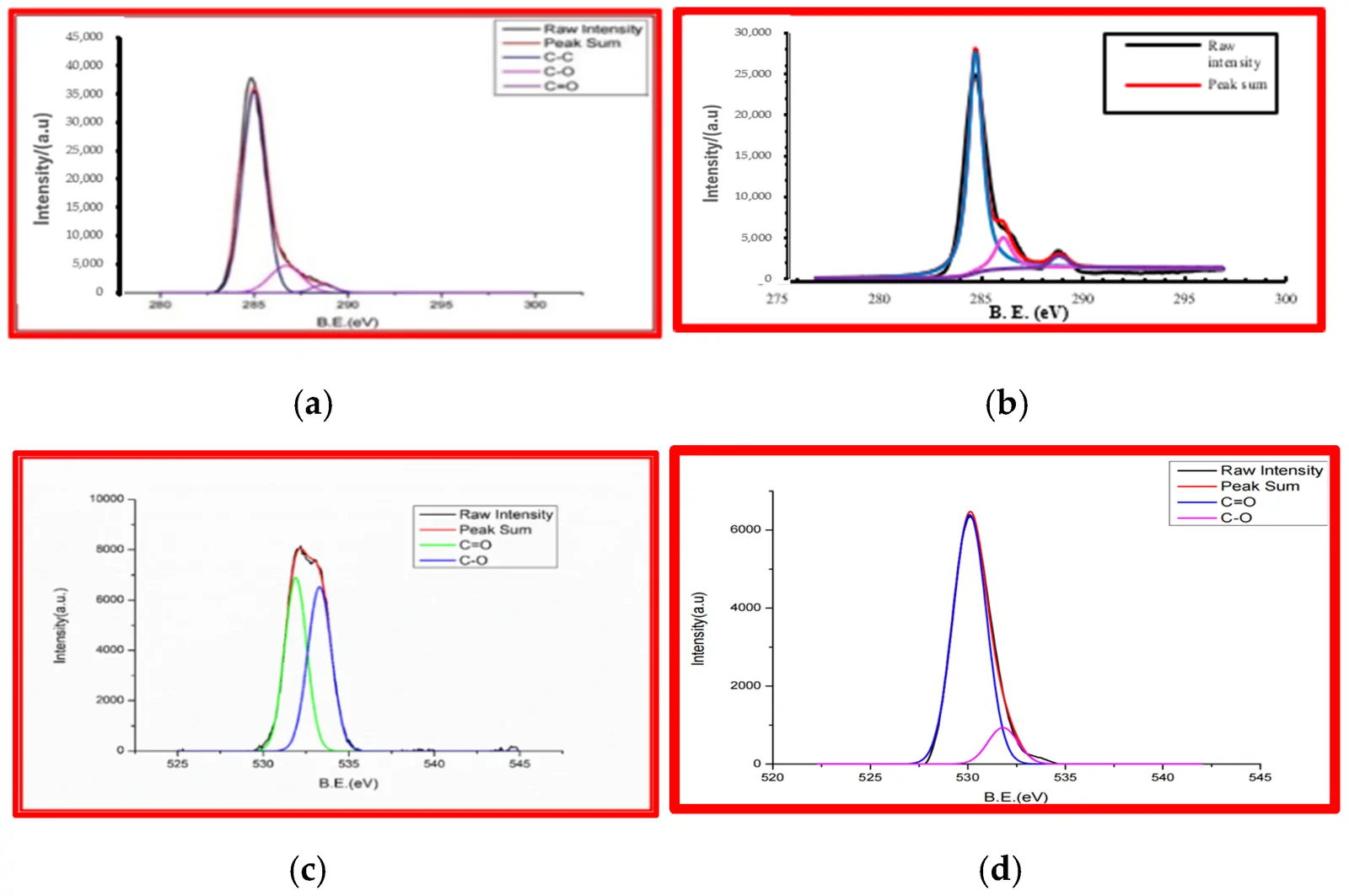
C1s peak of the XPS spectra of (**a**) untreated and (**b**) HCD plasma-treated PET tubes, and O1s peak of the XPS spectra of (**c**) untreated and (**d**) HCD plasma-treated PET tubes.

XPS was applied to study the chemical binding status of the internal surfaces for PET, PU, and PTFE tubes, before and after HCD plasma treatment. The comparison of XPS spectra between the untreated PET and the plasma-treated PET surfaces is shown in Figure 11. For the center part of the samples (Sample #5) placed in the chamber but without plasma generated internally, the XPS spectra are similar to the untreated sample. Table 7 summarizes the concentrations of C, O, and N signals obtained from the spectra of untreated and treated PET surfaces. According to this table, plasma treatment on the internal surfaces of PET tubes leads to a decrease in C concentration from 74.4 at. % to 61.9 at. %. At the same time, the plasma causes an increase in O concentration on the PET surface from 22.6 at. % to 35.6 at. %. Table 7 also shows that a small amount of nitrogen was found on the surface after plasma treatment. This is evidence of the existence of air molecules which were not pumped out thoroughly. From Table 7, it can be observed that the O/C ratio matches well with the wettability of the internal surfaces. The increase in O/C ratio after the HCD plasma treatment was accompanied by the decrease in WCA. To explain the relationship between wettability and O/C ratio, the deconvolution of the C1s peak obtained from the untreated PET sample was carried out. Three Gaussian–Lorentzian curves were observed. These peaks match well with three chemical bonds, i.e., C–C(H), C–O, and O–C=O. They are located at binding energies of 285.0 (C1), 286.5 (C2), and 289.0 (C3) eV, respectively [59,60,61].

After the HCD plasma treatment, the component (C1) is observed to decrease. The intensities of components (C2) and (C3) are observed to increase, respectively. The increased concentration of (C3) components is usually attributed to the enhancement of O-C=O groups. The O-C=O group is known to have polar behavior. Thus, a higher concentration of these polar groups can result in higher surface energy with lower WCA. The O1s peak was deconvoluted into two peaks, as shown in Figure 11c,d. The peaks at 531.9 (O1) eV and 533.5 (O2) eV are assigned to C=O and C-O bonds [59,62,63], respectively. After the HCD plasma treatment, component O1 increased significantly, whereas component O2 decreased. This finding is consistent with the deconvolutions of the C1s peak when O-C=O groups contribute to the formation of C3. Accordingly, an increase in O1, which is related to the C=O bond, could be expected. In sum, it is interesting to understand that WCA is correlated to the concentration of oxygen near the surface region as analyzed by XPS. As reported by many studies, it shows that an increased concentration of oxygen near the surface region reflects a lower WCA, thus better wettability. Furthermore, the increase in C=O intensity stands for the restructuring of the soft segment–ether group [62,63]. This is closely related to the reactions of O radicals originating from air. The increase in C=O intensity is hence driven by the production of O radicals. In sum, the plasma-treated surface is attributed to the restructuring of the ether group and the subsequent oxidation.

**Figure 12:**
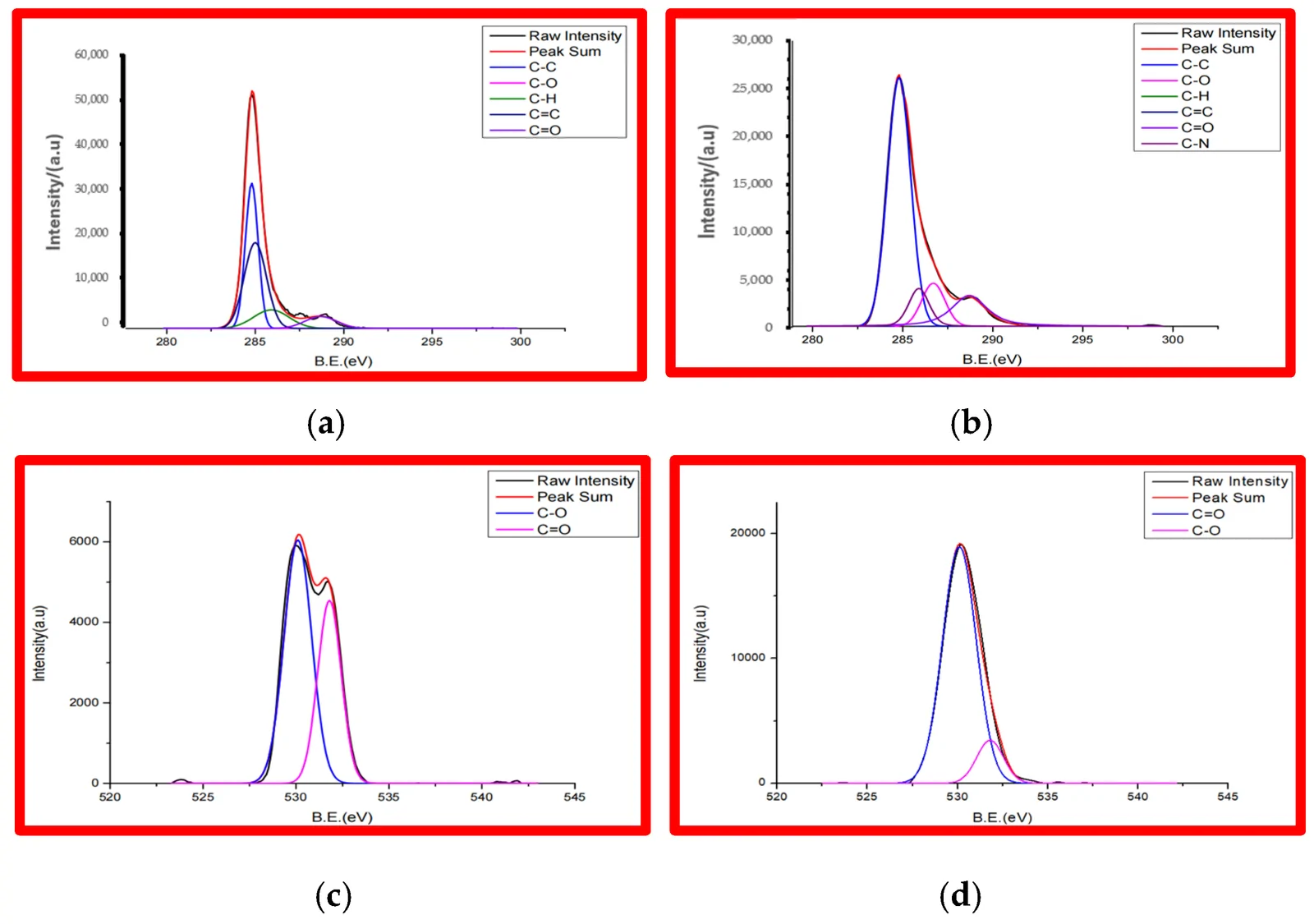
C1s peak of the XPS spectra of (**a**) untreated and (**b**) HCD plasma-treated PU tubes, and O1s peak of the XPS spectra of (**c**) untreated and (**d**) HCD plasma-treated PTFE tubes.

The XPS spectra for the untreated PU and the HCD plasma-treated PU are shown in Figure 12. Table 8 summarizes the surface concentrations of C, O, and N obtained from the spectra of untreated and HCD plasma-treated PU inner surfaces. The PU has aromatic and aliphatic double-bond hydrocarbons (–C=C, =C–H), carbonyl groups with different vibrations based on their conjugation (–N–C=O and O–C=O, respectively), an ether group (C–O–C), and N-containing groups in the fingerprint bonds (–NH) [28]. According to Table 8, it is seen that C1s concentrations decreased from 87.2% to 74.7%, O1s increased from 8.8% to 20.9%, and N1s did not change much after HCD plasma treatment. Figure 12a,b show that C-O and C=O increased significantly after HCD plasma treatment. The intensities of C=O and C-O also increased according to the perspective peaks of O1s, especially the C=O peak.

In contrast to the condition in conventional plasma treatment [28], the positive ions from the HCD plasma would break the bonds in the polymer surface, which would result in the enhancement of C=O bonds. Similarly, this can be explained with the restructuring of the ether group in soft segments due to the breakage of C bonds and the reaction with O radicals, concurrently with decreasing C-O intensity. This is the result of the direct effect of intensified ion bombardment in HCD plasma. These XPS results match well with the WCA measurements. It was found that low WCA was obtained from PU samples treated with HCD plasma. This implies the formation of new polar functional groups, which are rich with C=O bonds [64].

**Figure 13:**
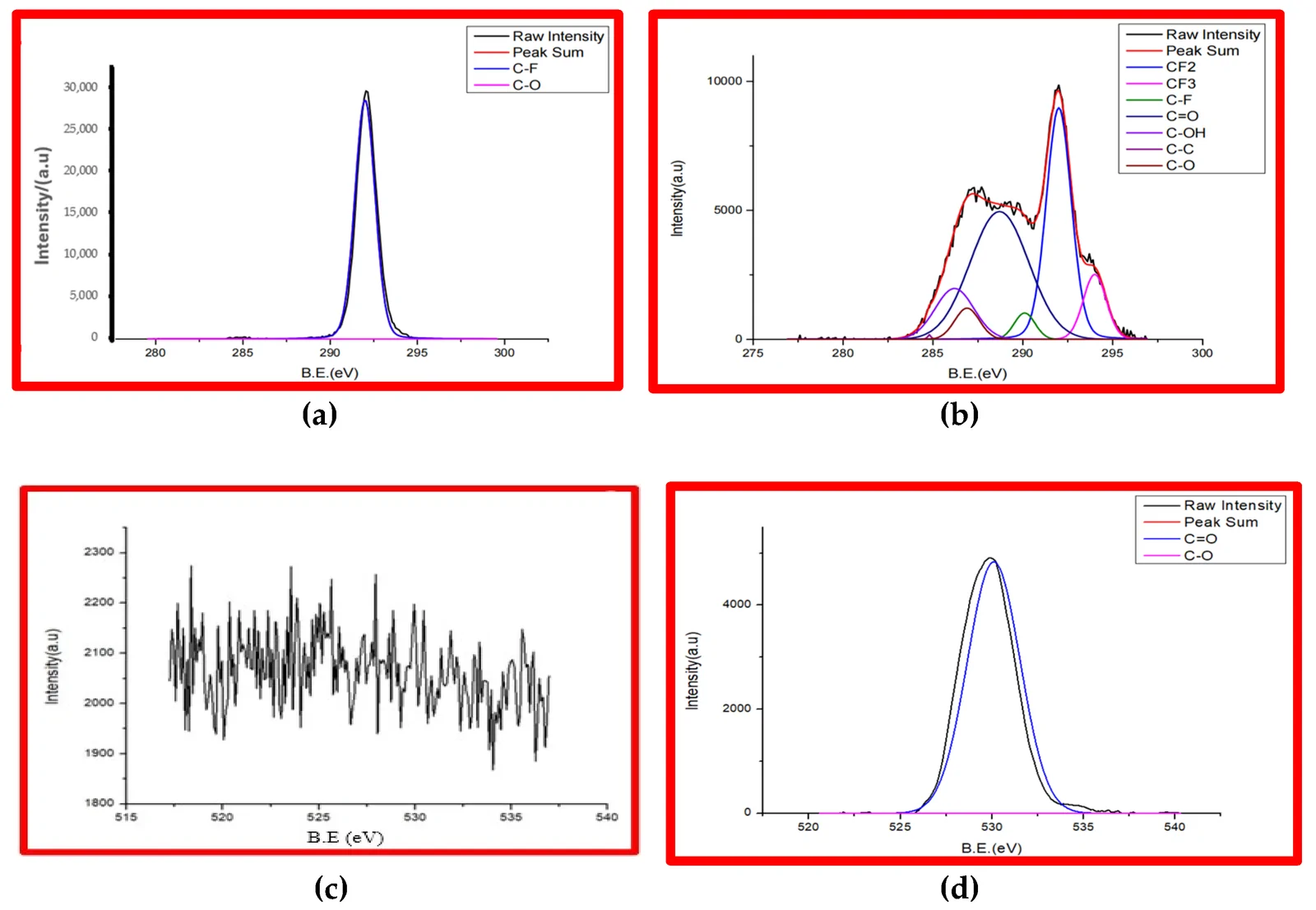
C1s peak of the XPS spectra of (**a**) untreated and (**b**) HCD plasma-treated PTFE tubes, and O1s peak of the XPS spectra of (**c**) untreated and (**d**) HCD plasma-treated PTFE tubes.

The comparison of XPS spectra for the untreated PTFE and the HCD plasma-treated PTFE is shown in Figure 13. Table 9 summarizes the surface concentrations of C, O, F, and N obtained from the spectra of untreated and HCD-treated PTFE surfaces. According to the XPS results (Table 9), it is observed that the F1S concentrations decreased from 66.7% to 41.7%, C1s increased from 33.3% to 47.4%, and O1s increased from <0.1% to 8.2% after HCD treatment. In Figure 13a,b, after peak deconvolution, it can be found that C-OH and C-C appear after HCD plasma treatment, respectively, and C-F, C-F2, and C-F3 all have a downward trend, and the O1s peak appears from noise to an obvious peak, as shown in Figure 13d.

For HCD plasma treatment of PTFE, the mechanisms governing the surface activation are still not clear [29]. Basically, it has been known that at least a moderate concentration of oxygen functional groups formed near the PTFE surface region is essential for better wettability. This is like other polymers. To date, it is certain that the activation of PTFE should be attributed to the attachment of foreign materials onto the surface. In HCD plasma treatment, this kind of attachment is achievable by inducing heavy ion bombardment on the surface since HCD plasma contains high-density ions. The bombardment of ions may cause bond breakage and thus the attachment of extra atoms with high surface energy. In addition, VUV radiation may be critical to the treatment of PTFE polymers with plasma. It was reported that the reaction ratio between VUV and reactive O species may be a decisive factor for the surface wettability [65]. In this regard, HCD plasma can provide high-density ion bombardment and intensified VUV irradiation.

Besides all the above-mentioned, ion bombardment on polymer may cause a change in micro-roughness, which may affect WCA. This can occur on almost any polymer treated with plasma, due to reactive ion etching. The change in micro-roughness may be particularly enhanced with high-density ion bombardment, such as HCD. Therefore, the influences of HCE on WCA should also include the change in micro-roughness, although this is not discussed in this study. The improvement of wettability should be the combined effects of physical (i.e., micro-roughness) and chemical (i.e., composition) modification. Both are related to HCD plasma density.

## 4. Conclusions

The purpose of this study is to investigate how HCD plasma can be generated and modify the inner walls of three polymer tubes, namely, PET, PU, and PTFE. A hollow-cathode model was proposed and proved to be able to explain the generation of plasma discharges inside the tubes. The tubes’ inner surfaces are hence activated. It was confirmed that HCD plasma can be generated inside the tube if the inner diameter of the tube is greater than twice the plasma sheath. This also explains the disappearance of plasma inside the tube with smaller diameters. Overall, the results show that when plasma density increases, the thickness of the sheath decreases. Accordingly, tubes with smaller diameters can be treated. When HCD is generated inside the polymer tubes, the surface becomes hydrophilic, which is partly attributed to the increase in carbon–oxygen bonds, particularly C=O bonds. This is not normally observed in the activation of polymers using CCP. A reasonable explanation is that HCE may enhance bond breaking by significant ion bombardment, together with radical oxidation. Also, although it is not studied, the change in micro-roughness due to high ion bombardment in HCD should not be ruled out as one of the reasons for decreasing WCA.

## Figures and Tables

**Table 1:** Examples of surface activation on various polymers with conventional capacitively coupled plasma (CCP) and unconventional high-density plasma technologies.

Processes	Plasma Process	Materials	Sample Shape	Results	Remarks
Conventional CCP for three materials	CCP RF plasma	PET	Film	WCA decreased to 36° by Ar + O_2_ plasma	APP treatment on PET substrate is more effective than CCP in terms of its wettability and defect density [40].
	CCP RF plasma	PU	Film (50 × 10 mm)	WCA decreased to 33° by air plasma	The most effective gas in increasing wettability was air, compared with that of the O_2_, N_2_, Ar, or their mixtures. The highest wettability was achieved with treatment for 180 s. This result was proved by the maximum oxygen content [28].
	CCP RF plasma	PTFE	Film (20 × 40 mm)	WCA decreased to 54° by oxygen plasma.	Oligomeric segments were formed due to the evolution of scission of the (CF_2_)n chain. This was caused by etching and decomposition of PTFE surface layer. Eventually, oxygen atoms should be incorporated into PTFE surface [41].
Unconventional high ion density processes	ICP plasma	PTFE	Foil (25 × 9 mm)	WCA decreased to 5° by successive H_2_ and O_2_ plasmas treat	The treatment with H_2_ plasma causes F depletion. Oxygen radicals in the afterglow region may cause functionalization of the H_2_-treated surface with polar functional groups. The highest surface energy was achieved in a limited range of O concentration [42].
	DC Plasma and Fast Neutrals (Ar)	PTFE	Tape (100 × 100 mm)	Reduction in WCA to 13°	Key advantages of the approach studied are the absence of fast ions. No charging effects occurred. It can run stably at low power levels. It can minimize surface damage and physical sputtering [7].
	Ion beam process	PC	Plate	WCA was changed from 78° to 12°	C=O bond percentage can increase from 3 to 17% after the Ar+ irradiation, which means that Ar ions were the main reason that caused chain breakage of C-C bonds. It is also attributed to the formation of more C=O groups due to the existence of oxygen in the processing chamber [43].
	Bipolar pulsed argon plasma	PC	Films of thickness 275 μm with sizes of 20 mm × 20 mm	WCA was changed from 63° to 17°	It was found that C-O and C=O functional groups would increase. The energy transfer may cause significant ion bombardment on the substrate surface. This is attributed to pulse action (high current density) [44].

**Table 2:** Material characteristics of the tested tube.

Characteristics	Tube Materials		
	PET	PU	PTFE
Family	polyester	Alternating copolymers	fluoropolymer
Aromatic ring	√	√ (with aliphatic structure)	
C-F bonds		√	
Surface energy /mJm^−2^	~52 [49]	~41.4 [50]	~22 [51]

**Table 3:** Process parameters for the verification of plasma generation inside the tubes’ material characteristics of the tested tubes.

Parameters	Range of Values
Power (Watt, RF)	70~260
Tube’s internal diameter (mm)	3, 4, 5, 6, 7
Tube’s length (cm)	10
Pressure (torr)	0.1, 0.4
Materials	PET, PU, PTFE

**Table 4:** Process parameters for the modification of the tubes’ internal surfaces.

	Process Parameters
Power (Watt, RF)	150, 260
Tube’s internal diameter (mm)	3, 4
Tube’s length (cm)	10, 15
Pressure (torr)	0.4
Addition of oxygen (sccm)	0, 5
Materials	PET, PU, PTFE

**Table 5:** *T_e_* and *n_e_*, as well as sheath thickness, as a function of RF power after calculation (Pressure = 0.1 torr).

0.1 torr (Ar)	Electron Temp. (eV), T_e_	Electron Density (cm^−3^), N_e_	Debye Length (mm), λ_D_	Plasma Sheath (mm), D_s_	T_e_/D_s_ (eV/mm)
70 W	0.638	2.60 × 10^11^	0.0116	3.34	-
90 W	0.626	2.71 × 10^11^	0.0112	3.24	-
150 W	0.619	3.70 × 10^11^	0.0096	2.76	0.224
200 W	0.604	4.54 × 10^11^	0.0085	2.46	0.246
220 W	0.598	5.26 × 10^11^	0.0080	2.36	0.254
260 W	0.581	5.78 × 10^11^	0.0076	2.04	0.285

**Table 6:** *T_e_* and *n_e_*, as well as sheath thickness, as a function of RF power after calculation (Pressure = 0.4 torr).

0.4 torr (Ar)	Electron Temp. (eV), T_e_	Electron Density (cm^−3^), N_e_	Debye Length (mm), λ_D_	Plasma Sheath (mm),D_s_	T_e_/D_s_ (eV/mm)
70 W	0.659	3.13 × 10^11^	0.0010	3.25	-
90 W	0.603	5.19 × 10^11^	0.0008	2.41	0.250
150 W	0.598	5.27 × 10^11^	0.00079	2.38	0.251
200 W	0.590	6.06 × 10^11^	0.00073	2.2	0.268
220 W	0.577	6.21 × 10^11^	0.00071	2.16	0.267
260 W	0.568	7.58 × 10^11^	0.00068	1.94	0.293

**Table 7:** Inner surface concentrations of C, O, and N before and after HCD plasma treatment on PET tubes.

	C1s	O1s	N1s
Untreated	74.4%	22.6%	<0.1%
HCD plasma	61.9%	35.6%	2.5%

**Table 8:** Inner surface concentrations of C, O, and N before and after HCD plasma treatment on PU tubes.

	C1s	O1s	N1s
Untreated	87.2%	8.8%	4.0%
HCD plasma	74.7%	20.9%	4.4%

**Table 9:** Inner surface concentrations of F, C, O, and N before and after HCD plasma treatment on PTFE tubes.

	F1s	C1s	N1s	O1s
Untreated	66.7%	33.3%	<0.1%	<0.1%
HCD plasma	41.7%	47.4%	2.8%	8.2%

## Data Availability

The data presented in this study are available on request from the corresponding author.

## References

[1] Li R., Ye L., Mai Y.W. (1997). Application of plasma technologies in fibre-reinforced polymer composites: A review of recent developments. Compos. Part A Appl. Sci. Manuf..

[2] Lohbach C., Bakowsky U., Kneuer C., Jahn D., Graeter T., Schafers H.J., Lehr C.M. (2002). Wet chemical modification of PTFE implant surfaces with a specific cell adhesion molecule. Chem. Commun..

[3] Tallarico M., Meloni S.M., Troia M., Cacciò C., Lumbau A.I., Gendviliene I., Ceruso F.M., Pisano M. (2025). The Use of Vacuum Plasma Surface Treatment to Improve the Hydrophilicity andWettability of Bone Graft Substitutes and Resorbable Membranes: An In Vitro Study. Dent. J..

[4] Fozza A.C., Klemberg-Sapieha J.E., Wertheimer M.R. (1999). Vacuum ultraviolet irradiation of polymers. Plasmas Polym..

[5] Fang Z., Qiu Y., Luo Y.J. (2003). Surface modification of polytetrafluoroethylene film using the atmospheric pressure glow discharge in air. J. Phys. D Appl. Phys..

[6] Sarani A., De Geyter N., Nikiforov A.Y., Morent R., Leys C., Hubert J., Reniers F. (2012). Surface modification of PTFE using an atmospheric pressure plasma jet in argon and argon+CO_2_. Surf. Coat. Technol..

[7] Komlev A., Kudryavtseva D., Neustroev I., Sudalenko Y., Altynnikov A., Tsymbalyuk A., Gagarin A., Platonov R. (2025). Improved Adhesion of PTFE Surfaces via Low-Power DC Plasma and Fast Neutrals Flow. Coatings.

[8] Lackner J.M., Kahn M., Waldhauser W. (2011). Plasma modification and deposition on inner tube faces by pulsed DC discharges. Vacuum.

[9] Li S., Huang W., Zhang J., Wang D. (2009). Optical diagnosis of an argon/oxygen needle plasma generated at atmospheric pressure Available to Purchase. Appl. Phys. Lett..

[10] Ionita E.R., Ionita M.D., Stancu E.C., Teodorescu M., Dinescu G. (2009). Small size plasma tools for material processing at atmospheric pressure. Appl. Surf. Sci..

[11] Schnabel U., Maucher T., Kohnlein J., Volkwein W., Niquet R., Trick I., Stieber M., Müller M., Werner H.P., Ehlbeck J. (2012). Multicentre trials for decontamination of fine-lumen PTFE tubes loaded with bacterial endospores by Low and atmospheric pressure. Plasma Process. Polym..

[12] Zhianmanesh M., Gilmour A., Bilek M.M.M., Akhavan B. (2023). Plasma surface functionalization: A comprehensive review of advances in the quest for bioinstructive materials and interfaces. Appl. Phys. Rev..

[13] Sham M.L., Lam M., Kim J.K. Adhesion characteristics of underfill material with various package components after plasma and UV/ozone treatments. Proceedings of the Advances in Electronic Materials and Packaging.

[14] Getty J.D., Zhao J. Plasma treatment for underfill process in flip chip packaging. Proceedings of the Pan Pacific Symposium.

[15] Egitto F.D., Emmi F., Horwath R.S., Vukanovic V. (1985). Plasma-etching of organic materials. 1. Polyimide in O_2_-CF_4_. J. Vac. Sci. Technol. B..

[16] Mittal K.L. (1977). The role of the interface in adhesion phenomena. Polym. Eng. Sci..

[17] Friedrich F., Mix R., Kühn G. (2003). Functional groups bearing plasma homo and copolymer layers as adhesion promoters in metal–polymer composites. Surf. Coat. Technol..

[18] Förch R., Zhang Z., Knoll W. (2005). Soft plasma treated surfaces: Tailoring of structure and properties for biomaterial applications. Plasma Process. Polym..

[19] Taheri S., Cavallaro A., Christo S., Majewski P.J., Barton M., Hayball J.D., Vasilev K. (2015). Antibacterial plasma polymer films conjugated with phospholipid encapsulated silver nanoparticles. ACS Biomater. Sci. Eng..

[20] Bhatt S., Pulpytel J., Mirshahi M., Arefi-Khonsari F. (2013). Plasma co-polymerized nanocoatings—As a biodegradable solid carrier for tunable drug delivery applications. Polymer.

[21] Li H., Chen S. (2015). Biomedical coatings on polyethylene terephthalate artificial ligaments. J. Biomed. Mater. Res. A.

[22] Li P., Cai X., Wang D., Chen S., Yuan J., Li L., Shen J. (2013). Hemocompatibility and anti-biofouling property improvement of poly(ethylene terephthalate) via self-polymerization of dopamine and covalent graft of zwitterionic cysteine. Colloids Surf. B.

[23] Sousa Coelho D., Veleirinho B., Alberti T., Maestri A., Yunes R., Dias P., Maraschin M., Clichici S., Filip A., Nascimento G.M.D. (2018). Electrospinning Technology: Designing Nanofibers toward Wound Healing Application. Nanomaterials—Toxicity, Human Health and Environment.

[24] Liu X., Xu H., Zhang M., Yu D.-G. (2021). Electrospun medicated nanofibers for wound healing: Review. Membranes.

[25] Liu Y., Zhou S., Gao Y., Zhai Y. (2019). Electrospun nanofibers as a wound dressing for treating diabetic foot ulcer. J. Pharm. Sci..

[26] Ng C., Hamzah M., Razak S.I.A., Sukor J., Nayan N., Hasraf N. (2020). Study on morphological properties of polyvinyl alco-hol/poly(lactic acid) wound dressing membrane as drug delivery carrier in wound healing treatment. J. Adv. Manuf. Technol..

[27] Pilehvar-Soltanahmadi Y., Dadashpour M., Mohajeri A., Fattahi A., Sheervalilou R., Zarghami N. (2018). An overview on ap-plication of natural substances incorporated with electrospun nanofibrous scaffolds to development of innovative wound dressings. Mini-Rev. Med. Chem..

[28] Abusrafa A.E., Habi S., Popelk A. (2020). Surface Functionalization of a Polyurethane Surface via Radio-Frequency Cold Plasma Treatment Using Different Gases. Coatings.

[29] Primc G. (2020). Recent Advances in Surface Activation of Polytetrafluoroethylene (PTFE) by Gaseous Plasma Treatments. Polymers.

[30] Golda J., Biskup B., Layes V., Winzer T., Benedikt J. (2020). Vacuum ultraviolet spectroscopy of cold atmospheric pressure plasma jets. Plasma Process. Polym..

[31] Pessoa R.S., Sismanoglu B.N., Amorim J., Maciel H.S., Petraconi G., Kerala J.A. (2007). Gas Discharges: Fundamentals and Applications.

[32] Bárdŏs L. (1996). Radio frequency hollow cathodes for the plasma processing technology. Surf. Coat. Technol..

[33] Bardos L., Barankova H., Berg S. (1997). Thin film processing by radio frequency hollow cathodes. Surf. Coat. Technol..

[34] Baguer N., Bogaerts A., Gijbels R. (2003). Role of the fast Ar atoms, Ar+ ions, and metastable Ar atoms in a hollow cathode glow discharge: Study by a hybrid model. J. Appl. Phys..

[35] Baguer N., Bogaerts A., Donko Z., Gijbels R., Sadeghi N. (2005). Study of the Ar metastable atom population in a hollow cathode discharge by means of a hybrid model and spectrometric measurements. J. Appl. Phys..

[36] Baguer N., Bogaerts A. (2005). Study of the sputtered Cu atoms and ions in a hollow cathode glow discharge using a hybrid model. J. Appl. Phys..

[37] Kirkici H., Bruno D. (1995). Operating characteristics of a segmented hollow cathode over a wide pressure range. IEEE Trans. Plasma Sci..

[38] Carbone E.A.D., Verhoeven M.W.G.M., Keuning W., van der Mullen J.J.A.M. (2016). PTFE treatment by remote atmospheric Ar/O_2_ plasmas: A simple reaction scheme model proposal. J. Phys. Conf. Ser..

[39] Ryu J., Kim K., Park J., Hwang B.G., Ko Y., Kim H., Han J., Seo E., Park Y., Lee S.J. (2017). Nearly Perfect Durable Superhydrophobic Surfaces Fabricated by a Simple One-Step Plasma Treatment. Sci. Rep..

[40] Prakash A., Manohara K., Kumar S., Katiyar M. (2025). Effect of plasma processing on the morphological and electrical properties of polyethylene terephthalate (PET) substrate. Results Surf. Interfaces.

[41] Liu C.Z., Wu J.Q., Ren L.Q., Tong J., Li J.Q., Cui N., Brown N.M.D., Meenan B.J. (2004). Comparative study on the effect of RF and DBD plasma treatment on PTFE surface modification. Mater. Chem. Phys..

[42] Lojen D., Zaplotnik R., Primc G., Mozetič M., Vesel A. (2020). Effect of VUV radiation and reactive hydrogen atoms on depletion of fluorine from polytetrafluoroethylene surface. Appl. Surf. Sci..

[43] Cho J.S., Koh S.K. (2000). Enhanced adhesion between polycarbonate substrate and tin doped indium oxide films by ion assisted reaction. Nucl. Instrum. Methods. Phys. Res. B.

[44] Pelagade S., Singh N.L., Shah S., Qureshi A., Rane R.S., Mukherjee S., Deshpande U.P., Ganesan V., Shripathi T. (2010). Surface free energy analysis for bipolar pulsed argon plasma treated polymer films. J. Phys. Conf. Ser..

[45] Mishra H., Bolouki N., Hsieh S., Li C., Wu W., Hsieh J.H. (2022). Application of Spectroscopic Analysis for Plasma Polymerization Deposition onto the Inner Surfaces of Silicone Tubes. Coatings.

[46] Vazquez F.J.G., Camero M., Aleixandre C.G. (2006). Spectroscopic measurements of the electron temperature in low pressure radiofrequency Ar/H_2_/C_2_H_2_ and Ar/H_2_/CH_4_ plasmas used for the synthesis of nanocarbon structures. Plasma Sources Sci. Technol..

[47] Zhu X.M., Chen W.C., Li J., Pu Y.K. (2009). Determining the electron temperature and the electron density by a simple collisional radiative model of Ar and xenon in low pressure discharge. J. Phys. D Appl. Phys..

[48] Lindner M. (2020). Effect of Substrate Strain, Aluminum Thickness and Corona Pretreatment on the Electrical Resistance of Physical Vapor Deposited Aluminum Coatings. Coatings.

[49] Song X., Zhang X., Li T., Li Z., Chi H. (2019). Mechanically Robust Hybrid POSS Thermoplastic Polyurethanes with Enhanced Surface Hydrophobicity. Polymers.

[50] Comyna J., De Buyl F., Subramanian C. (2002). The effect of contact with PTFE tape on the moisture cure of an alkoxysilicone sealant. Int. J. Adhes. Adhes..

[51] Grill A. (1994). Cold Plasma Materials Fabrication: From Fundamentals to Applications.

[52] Muhl S., Pérez A. (2015). The use of hollow cathodes in deposition processes: A critical review. Thin Solid Film..

[53] Koh S.K., Choi S.C., Han S., Cho J., Choi W.K., Jung H.J. (1998). Surface modification of polymer by ion assisted reaction. Key Eng. Mater..

[54] Cho J.S., Han S., Kim K.H., Beag Y.W., Koh S.K. (2003). Surface modification of polymers by ion-assisted reaction. Thin Solid Film..

[55] Valerio J.K.C., Nakajima H., Vasquez M.R. (2019). Grafting of acrylic acid onto microwave plasma-treated polytetrafluoroethylene (PTFE) substrates. Jpn. J. Appl. Phys..

[56] Nguyen H.D., Yajima T. (2017). A Spectroscopic Study on Argon-Ammonia Water Gaseous Plasma Super-hydrophilizing Polytetrafluoroethylene. J. Photopolym. Sci. Technol..

[57] Chen F., Song J., Huang S., Xu S., Xia G., Yang D., Xu W., Sun J., Liu X. (2016). Simultaneous and long-lasting hydrophilization of inner and outer wall surfaces of polytetrafluoroethylene tubes by transferring atmospheric pressure plasmas. J. Phys. D Appl. Phys..

[58] Chen F., Liu S., Liu J., Huang S., Xia G., Song J., Xu W., Sun J., Liu X. (2016). Surface modification of tube inner wall by transferred atmospheric pressure plasma. Appl. Surf. Sci..

[59] Inagaki N., Narushim K., Tuchida N., Miyazaki K. (2004). Surface characterization of plasma-modified poly(ethylene terephthalate) film surfaces. J. Polym. Sci. Part B Polym. Phys..

[60] Groning P., Collaud M., Dietler G., Schlapbach L. (1994). Plasma modification of polymethylmethacrylate and polyethyleneterephthalate surfaces. J. Appl. Phys..

[61] Vesel A., Junkar I., Cvelbar U., Kovac J., Mozetic M. (2008). Surface modification of polyester by oxygen- and nitrogen-plasma treatment. Surf. Interface Anal..

[62] Kormunda M., Pavlik J. (2010). Characterization of oxygen and argon ion flux interaction with PET surfaces by in-situ XPS and ex-situ FTIR. Polym. Degrad. Stab..

[63] Gonzalez E., Barankin M.D., Guschl P.C., Hicks R.F. (2008). Remote Atmospheric-Pressure Plasma Activation of the Surfaces of Polyethylene Terephthalate and Polyethylene Naphthalate. Langmuir.

[64] Fu X., Jenkins M.J., Sun G., Bertoti I., Dong H. (2012). Characterization of active screen plasma modified polyurethane surfaces. Surf. Coat. Technol..

[65] Fantz U., Briefi S., Rauner D., Wünderlich D. (2016). Quantification of the VUV radiation in low pressure hydrogen and nitrogen plasmas. Plasma Sources Sci. Technol..

